# Alexithymia in Schizophrenia and Psychosis Vulnerability: A Systematic Review and Meta‐Analysis

**DOI:** 10.1002/jclp.23788

**Published:** 2025-03-19

**Authors:** Ercan Ozdemir, Zhuoni Xiao, Helen Griffiths, Angus MacBeth

**Affiliations:** ^1^ School of Health in Social Science, Section of Clinical Psychology University of Edinburgh Edinburgh UK; ^2^ Department of Psychology University of Edinburgh Edinburgh UK

**Keywords:** adult mental health, clinical psychology, emotion regulation

## Abstract

**Aims:**

Disturbances involving impairments in experience and expression of affect are frequently identified in schizophrenia samples. Alexithymia underlies cognitive impairments in identification and expression of affect, further implicated in affect dysregulation. The current review aimed to systematically review the literature and estimate the strength of associations between alexithymia and schizophrenia phenomenology.

**Method:**

A systematic review and meta‐analysis identified 67 studies involving measures of alexithymia in psychosis. All studies were assessed for quality and publication bias. Overall, data from 47 studies were suitable for meta‐analysis.

**Results:**

Alexithymia and schizophrenia were consistently positively associated with a large effect size (*k* = 11). Compared to control groups, a schizophrenia diagnosis was positively associated with large magnitude effects for difficulties in identifying feelings (*k* = 18) and moderate effect sizes for difficulties in describing feelings (*k* = 17) and externally oriented thinking (*k* = 11). Data from community samples indicated moderate associations between subclinical negative symptoms and difficulties in identifying and describing feelings (*k* = 4) and a small association between positive symptoms and difficulties in identifying feelings (*k* = 5).

**Conclusions:**

Alexithymia and schizophrenia are strongly associated. However, methodological issues limit the establishment of directionality in these associations. The majority of studies use cross‐sectional designs reliant on self‐report assessments which may result in over‐estimation of the reported effect sizes. Future research could conceptualize alexithymia as a stress‐reactive multidimensional construct, and modeling dynamic relationships between alexithymia, psychological distress, and schizophrenia phenomenology should incorporate confounders such as gender, age, and neurocognition.

## 1 Introduction

A disturbance of affectivity characterized by diminished experience of positive affect and elevated experience of negative affect has been described as a cardinal feature of schizophrenia (Bleuler 1950/1911). Indeed, a diagnosis of schizophrenia has been associated with elevated negative affect and diminished positive affect in individuals' daily lives compared to individuals without the diagnosis (Cho et al. 2017) and affect dysregulation has further been identified as a pathway to psychosis onset (Bogudzińska et al. 2024; Muddle et al. 2021; Myin‐Germeys and van Os 2007). Alexithymia refers to cognitive impairments in emotion processing conferring proneness to affect dysregulation (Preece et al. 2023; Taylor 2000). Accordingly, understanding alexithymia as a psychological factor is important, as it may constitute a mechanism underlying risk of psychosis development.

Current definitions of alexithymia comprise impairments in identifying and expressing affects and in imaginal capacity, coupled with externally oriented thinking (Taylor 2000). Alexithymia has been proposed to represent a set of trait impairments, the severity of which may become exacerbated in states of psychological distress (Taylor and Bagby 2013). Alexithymia is primarily assessed using self‐report measures, of which the Toronto Alexithymia Scale (TAS; Bagby et al. 1994) and the Bermond‐Vorst Alexithymia Questionnaire (BVAQ; Vorst and Bermond 2001) are the most commonly used assessments (Sekely et al. 2018).

In the TAS, alexithymia has been operationalized along dimensions of difficulties in identifying feelings (DIF), difficulties in describing feelings (DDF), and externally oriented thinking (EOT). The TAS paradigm traditionally classifies alexithymia categorically as a trait quality based on a cutoff point ( > 61) applied to the total scale score, although more recently a continuum approach applied to the total scale score has been favored (Bagby et al. 2020). Alexithymia as measured with the TAS has been observed to fluctuate in states of elevated distress (Honkalampi et al. 2018; Taylor et al. 2016), depression, and anxiety (Taylor and Bagby 2013; Li et al. 2015). In the BVAQ model, alexithymia has been conceptualized multidimensionally indicated by identifying, describing, and analyzing emotions, diminished fantasizing, and diminished emotional reactivity dimensions (i.e., emotionalizing; Vorst and Bermond 2001).

From a multidimensional perspective, differential associations have been suggested between different components of alexithymia and emotion processing respectively, such that EOT may be linked to attentional biases towards avoidance of emotional experiences, DDF may be involved in appraisal of emotional experiences, and DIF may be associated with negative affective reactivity (Luminet et al. 2021). Differential associations may be observed between schizophrenia phenomenology and multidimensional components of alexithymia based on the degree of conceptual overlap between the constructs, particularly for alogia and social anhedonia and DDF; and between DIF and thought disorder/schizotypal disorganization. Negative symptomatic experiences such as attenuated social relatedness and verbal expressivity may represent confounding factors in the expression of affective experiences. Similarly, identification of affects may be confounded by severity of distress due to unusual self‐experiences.

A previous meta‐analysis of affect regulation and dissociation in schizophrenia reported that alexithymia conceptualized unidimensionally had a highly heterogeneous moderate to large effect size for positive associations with schizophrenia, based on case‐control studies (O'Driscoll et al. 2014). Additionally, a systematic review of emotion regulation in psychosis implemented a multidimensional approach to alexithymia and suggested that the DIF and DDF dimensions were positively associated with psychosis, while the associations between EOT and psychosis were not explored (Lawlor et al. 2020). The current systematic review and meta‐analysis aimed to synthesize the existing research by investigating the relationship between schizophrenia and alexithymia as both a unidimensional and multidimensional research construct in the following domains: (1) assessment of the severity of unidimensional alexithymic impairment within schizophrenia samples, (2) investigation of subgroup differences in unidimensional alexithymia within schizophrenia population based on presence or absence of paranoia, (3) comparison of the Nonclinical control and schizophrenia groups on unidimensional and multidimensional alexithymia, and (4) exploration of the associations between multidimensional alexithymia and schizophrenia symptom dimensions.

## 2 Methods

**Figure 1:**
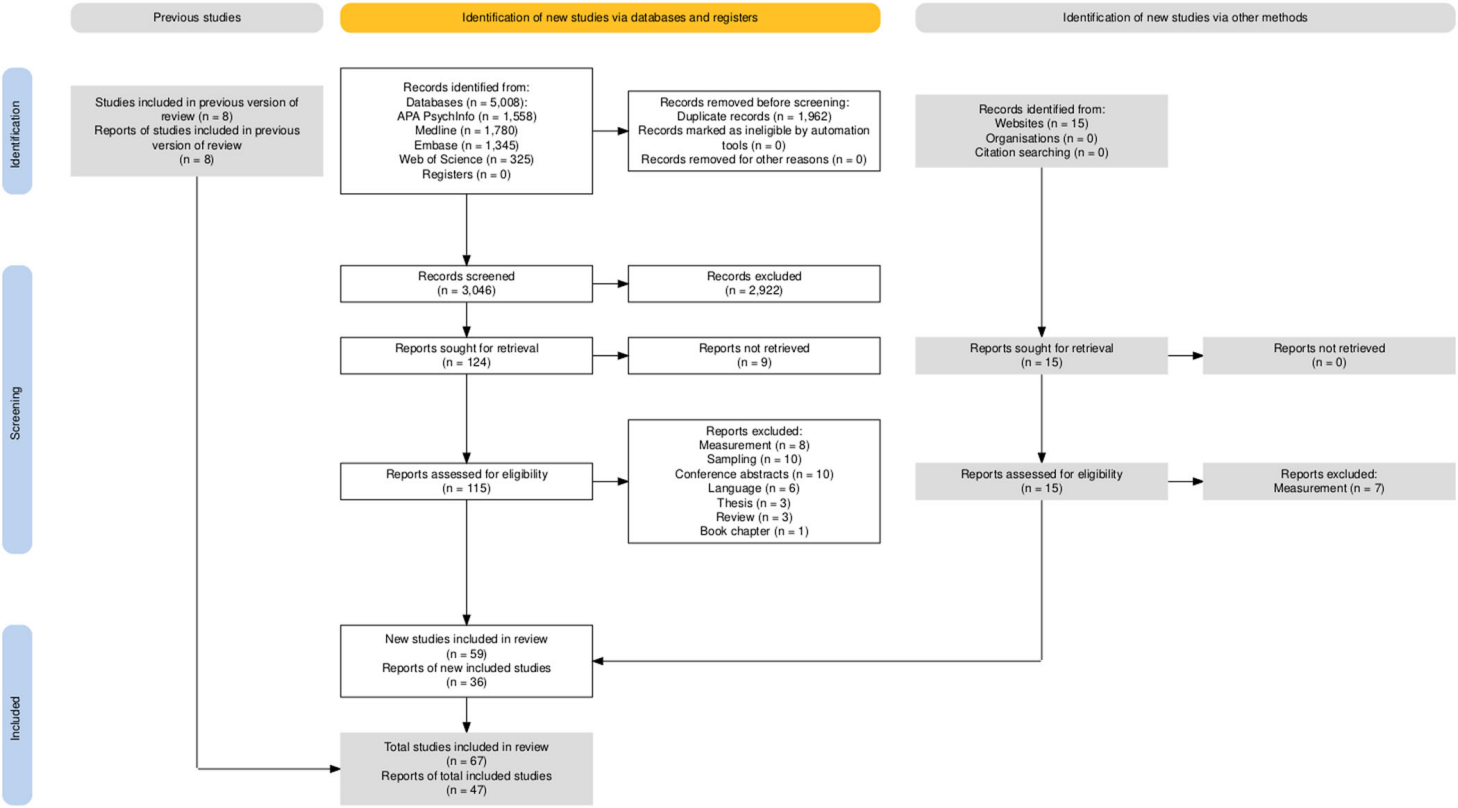
PRISMA flow diagram of the review process.

The review protocol was registered in PROSPERO (ID number CRD42022327375). Deviations from the registered protocol were: 1) The original research question, “What is the strength of the association between alexithymia and psychotic phenomenology, symptoms, and functioning?” was further elaborated in the four‐domain formulation of the research aims. 2) original exclusion criteria were extended to involve conference abstracts, review articles, meta‐analyses, theses and dissertations beyond qualitative and case studies. The PRISMA flow chart is displayed in Figure 1 (see Checklist S1).

### 2.1 Search Strategy

The search involved the following databases: PsycINFO, MEDLINE, Embase, and Web of Science. Google Scholar was searched to identify the articles that are not indexed in databases. The search was restricted to research articles, published after 1973 (when the concept of alexithymia was first articulated) and written or translated into English. Search terms combined psychosis OR psychoses OR psychotic OR schizo* OR paranoi* OR hallucinat* OR delusion* OR “ultrahigh risk” OR prodrom* OR “at risk mental state” AND Alexithymia OR “affective symptom*”. Each step of selection, quality assessment, and data extraction involved cross‐validation by authors EO and ZX.

### 2.2 Selection

Citations identified by the search were imported to Covidence for initial screening of titles and abstracts. Inclusion criteria involved peer reviewed articles using validated assessment tools that report quantitative data on:

- Psychotic experiences encompassing subclinical, prodromal or clinical forms of negative, disorganized, and positive symptoms,
- Alexithymia.

Exclusion criteria were:

- Qualitative studies, case studies, conference abstracts, review articles, meta‐analyses, theses and dissertations.

Articles potentially meeting the inclusion criteria were screened at full text level.

### 2.3 Data Extraction and Analyses

Extracted data included sample sizes and sample characteristics, mean, standard deviations, and correlation coefficients where available, which were inputted to an Excel sheet. A narrative synthesis was used to describe the main characteristics of each study and the R packages {meta} (Balduzzi et al. 2019), {dmetar} (Harrer et al. 2019), and {metafor} (Viechtbauer 2010) were used to conduct the meta‐analyses. Readers may consult the references for the R packages for a conceptual overview and practical applications of the conducted analyses. Meta analytic methods utilized inverse variance and Q‐Profile to estimate confidence intervals for tau^2^ and tau. Random effects models were adjusted using the Hartung‐Knapp method to calculate the confidence interval around the pooled effect.

Raw means and standard deviations were used as the parameter of central tendency for the meta‐analysis of means, quantifying alexithymia scores in schizophrenia samples relative to the Toronto Alexithymia Scale (TAS; Bagby et al. 1994) total cut‐off score of > 61. The restricted maximum‐likelihood estimator was used to estimate heterogeneity variance (τ^2^) based on the meta‐analysis of means. The Sidik‐Jonkman estimator was to be used if the restricted maximum‐likelihood estimator indicated substantial heterogeneity. Subgroup analyses were conducted on the meta‐analysis of means to compare samples of paranoid and non‐paranoid schizophrenia patients.

Random effects meta‐analyses of mean differences were conducted between schizophrenia and Nonclinical samples to compare uni‐ and multi‐dimensional alexithymia levels. In these analyses, the Sidik‐Jonkman estimator was used to assess heterogeneity and Hedges g to standardize the mean difference.

A meta‐analysis of correlations was conducted to pool the correlation coefficients between alexithymia dimensions and schizophrenic symptom dimensions, and restricted maximum‐likelihood estimators were used to calculate heterogeneity. The *metacor* function of the {meta} package was used for the correlational analyses. The procedure involved transforming raw correlation coefficients using Fisher's *z*‐transformation, which are then converted back to original form in the output.

Results were assessed for outlying and influential studies. The “find. outliers” function in the {dmetar} package was used to detect extreme individual studies' effect sizes compared to the overall effect. Influence diagnostics were calculated based on leave‐one‐out method and were examined using Baujat, diagnostics, overall effect, and *I* ^2^ heterogeneity plots. Results of the sensitivity analyses excluding the outlying and/or influential cases are reported.

Moderator analyses were conducted on the studies included in the sensitivity analyses. Alexithymic impairment was reported to increase with age (Mattila et al. 2006), which was included as a moderator alongside study quality. Publication bias was visually assessed using funnel plots and statistically assessed using Eggers' test.

### 2.4 Quality Assessment

The Newcastle‐Ottawa Quality Assessment Scale for case‐control studies (Wells et al. 2014) was used to assess study quality. The maximum score that the scale generates is 9 divided into three categories: selection (four points), comparability (two points), and exposure (three points). The selection category involved four criteria: adequacy of case definition, representativeness of cases, selection of controls, and definition of controls. The comparability category involved one subsection and the assignment of points is based on analyses of potential covariates. Two of the exposure criteria evaluated quality of assessment and the third criterion evaluated attrition and missing data handling. Three quality categories can be generated from the 9‐point scale ranging from poor (a score of less than 3), fair (a score between 3 and 5), and good/high (a score between 6 and 9).

## 3 Results

### 3.1 Study Characteristics

Among the 67 included studies, 1 used an experience sampling design, 5 were interventions, and 61 utilized cross‐sectional designs. Baseline measurements were retrieved from 6 studies that used repeated measures designs (Caccamo et al. 2019; Chung et al. 2013; Hsu and Ouyang 2021; Metzner et al. 2018; Uzun and Lok 2022), one of which was a momentary assessment study (Kimhy et al. 2020). Forty‐nine studies involved clinical samples (*N* = 2823, 38% female), of which 29 studies used case‐control designs (n_case_ = 1316, n_control_ = 1264), and 18 studies involved community samples (*N* = 5817, 60% female). For the community studies, 45% of the overall participants belonged to one study (Ma et al. 2020). The mean age of participants in clinical studies was 37.08 years and 22.15 years in community studies. The majority of the studies were from Europe (*n* = 28), followed by the USA (*n* = 19), East Asian (*n* = 12), Middle Eastern (*n* = 4) countries, South Asia (*n* = 2), and Australasia (*n* = 1). The Toronto Alexithymia Scale (Bagby et al. 1994) was used in 55 studies and the Bermond‐Vorst Alexithymia Questionnaire (Vorst and Bermond 2001) in 11 studies.

Clinical studies using formal diagnosis utilized DSM‐III‐R (*n* = 1), DSM‐IV (*n* = 33), DSM‐V (*n* = 5), ICD‐10 (*n* = 6), and the MINI (*n* = 1), whilst 4 studies did not use a formal diagnostic method. Studies reporting homogenous diagnoses involved schizophrenia (*n* = 21), paranoid schizophrenia (*n* = 4), high‐risk of psychosis (*n* = 2), and first episode psychosis (*n* = 1) samples. Nineteen studies involved heterogeneous samples: 6 samples predominantly included paranoid schizophrenia patients (*n* > 50%), 9 studies reported diagnoses across the schizophrenia spectrum (e.g., schizophrenia, schizoaffective, schizophreniform), and 3 studies primarily involved bipolar or mood disorders (*n* > 50%) (Caccamo et al. 2019; Picardi et al. 2012; Solano et al. 2000).

### 3.2 Quality Assessment

The mean quality assessment score was 3.51 (SD = 2.35; Range = 1 to 7). Twenty‐three studies were rated to have poor quality, 25 studies were classified as showing fair quality and 19 studies showed good quality, indicating overall issues with quality.

### 3.3 Narrative Synthesis

#### 3.3.1 Alexithymia in Schizophrenia

Studies comparing schizophrenia to healthy control samples reported higher alexithymia scores in schizophrenia samples than in controls (Heshmati et al. 2010; Koelkebeck et al. 2010; Kubota et al. 2011, 2012; Lee et al. 2021). Comparisons between schizophrenia, healthy control and other diagnostic groups reported a more severe alexithymic profile in schizophrenia (Kumar et al. 2018; Torregrossa et al. 2022), anxiety disorder (Opoka et al. 2021), somatic disorders (Solano et al. 2000), and bipolar disorder (Ospina et al. 2019) compared to healthy control groups, although these diagnostic groups did not differ from each other in alexithymia profiles. However, significant differences were observed between schizophrenia and substance use disorder, with individuals in the schizophrenia sample reporting higher alexithymia scores (Lysaker et al. 2017).

Among psychotic disorders, non‐affective and affective psychoses did not differ in alexithymia levels (Picardi et al. 2012). However, non‐paranoid schizophrenia patients reported higher alexithymia scores than paranoid patients (Stanghellini and Ricca 1995), while alexithymia still predicted paranoia independently of negative emotions, perceptual anomalies and sleep (Rehman et al. 2018). Paranoid schizophrenia patients reported higher alexithymia scores compared to a healthy control group (Cedro et al. 2001) and alexithymia was associated with hallucinations but not delusions in a paranoid sample (Gawęda and Krężołek 2019). These findings suggest that although paranoid patients may have attenuated scores on alexithymia compared to non‐paranoid schizophrenia patients, alexithymia was still related to paranoia when multiple confounders were controlled for. After excluding Cedro et al.'s study (2001) the remaining studies obtained low quality scores.

Research reporting dimensional relationships between symptomatology and alexithymia was limited. Psychotic patients showed elevated difficulties in describing feelings compared to affective and severe personality disorders although general symptom severity in the psychosis sample was lower (Caccamo et al. 2019). Schizophrenia patients reported higher scores on the fantasizing dimension of alexithymia compared to an autism spectrum disorder sample (Hyatt et al. 2021). In one study apiece, difficulties identifying feelings were found to be associated with paranoia (Yu et al. 2011), pre‐delusional experiences (Maggini and Raballo 2004), and hallucinations (Serper and Berenbaum 2008). Positive associations between externally oriented thinking and neurocognitive deficits were reported in two studies (Fogley et al. 2014; Luo et al. 2021), whereas negative associations between externally oriented thinking and emotion recognition (Tang et al. 2016) and depression (He et al. 2022) were found in one study each.

### 3.4 Alexithymia in Psychosis Vulnerability

In terms of subclinical psychotic experiences, alexithymia (measured with the TAS) was strongly associated with schizotypy (Rus‐Calafell et al. 2013; Van 't Wout et al. 2004), magical thinking (Eddy and Hansen 2021), negative schizotypy (Koven 2014), and cluster A personality disorders (Dimaggio et al. 2011). Studies using the BVAQ reported elevated alexithymia for schizotypal personality disorder diagnosis compared to a Nonclinical control sample (Dickey et al. 2012), whilst another study found no association between alexithymia and schizotypy measured in a Nonclinical sample (Larøi et al. 2008). One study using an at risk for psychosis sample suggested that affective alexithymia, defined by low emotional reactivity and fantasizing, may be related to at risk states, negative and depressive symptoms (van der Velde et al. 2015). Affective alexithymia was also linked to subclinical hallucinatory experiences (Van 't Wout et al. 2004) and positive, negative, and disorganized schizotypal experiences (Larøi et al. 2008).

The most consistent dimensional associations were between difficulties identifying and describing feelings and negative schizotypal experiences (Larøi et al. 2008; Martin et al. 2016; Prince and Berenbaum 1993). There may be a pattern in the dimensional associations, such that subclinical negative symptoms may be more strongly related to difficulty in describing feelings and subclinical positive symptoms to difficulty in identifying feelings (Fung et al. 2017; van Rijn et al. 2011; Van 't Wout et al. 2004; Yang et al. 2020).

### 3.5 Covariates of Alexithymia

Based on small numbers of studies (1–3 studies for each outcome) alexithymia in psychotic experiences may be associated with earlier age of psychosis onset (Gawęda and Krężołek 2019; Son et al. 2012), suicide (De Berardis et al. 2013; Demirkol et al. 2019; Picardi et al. 2012), emphatic impairments (Aaron et al. 2015; Yang et al. 2020), emotion recognition problems (Rus‐Calafell et al. 2013) and social cognition deficits (Etchepare et al. 2019), proneness towards cognitive biases (Gawęda and Krężołek 2019), sleep problems (Ma et al. 2020), more intense psychotic experiences in immigrants (Pozza 2019), depersonalization (Maggini et al. 2002). Alexithymia was reported to be positively associated with negative symptoms (Tang et al. 2016; Ustundag et al. 2020) and a study assessing alexithymia's relationship to negative symptom dimensions reported a stronger association between alexithymia and alogia (Henry et al. 2010). Two studies, reported no associations between negative symptoms and alexithymia (Todarello et al. 2005; Valdés et al. 2008).

Depression has been reported as a covariate of alexithymia in schizophrenia (He et al. 2022; Maggini et al. 2004; Todarello et al. 2005; van der Meer et al. 2009; van der Velde et al. 2015). Higher alexithymia scores were reported by depressed vs nondepressed schizophrenia patients (Lysaker et al. 2013; Maggini et al. 2004) and that depression might be more related to difficulties in identifying (van der Meer et al. 2009) and describing feelings (He et al. 2022). Childhood traumatic neglect may be associated with difficulties in identifying feelings, in the context of schizotypy, at least in female samples (Berenbaum et al. 2003). One study reported neurocognitive deficits and anxiety as predictive of alexithymia (Fogley et al. 2014).

Emotional distress may be associated with elevated alexithymia scores: distress due to symptom severity was associated with alexithymia (Maggini et al. 2003; Picardi et al. 2012), heightened levels of emotional distress might be linked to difficulties in describing (Fogley et al. 2014) and identifying feelings (van 't Wout et al. 2007). Findings suggesting male schizophrenia patients were more liable to experience heightened difficulty in identifying feelings under emotional distress (van 't Wout et al. 2007) and had higher trait‐like difficulty in describing feelings compared to female schizophrenia patients (Gupta and Gupta 2019). These may implicate male gender as a risk factor for more severe alexithymia presentations.

### 3.6 Alexithymia and Psychosocial Functioning

Alexithymia may also adversely affect psychosocial functioning in schizophrenia. Psychometrically identified Alexithymia in schizophrenia patients was associated with lower functioning scores compared to non‐alexithymic patients (Ustundag et al. 2020) and alexithymia was found to be associated with social functioning in a community sample assessed on schizotypy (Seghers et al. 2011). Further, alexithymia was reported as a plausible mediator between cognitive impairment and functional outcome (Luo et al. 2021). Specific associations were found, in which functioning was predominantly linked to difficulty in describing feelings (Kimhy et al. 2012, 2016). However, these patterns of results were not observed in two studies (Ospina et al. 2019; Todarello et al. 2005), which reported that difficulty in describing feelings was only a predictor of functioning in bipolar disorder (Ospina et al. 2019); and negative symptoms, but not alexithymia, were associated with functioning (Todarello et al. 2005).

In terms of psychotherapeutic interventions, cognitive behavioral therapy (Chung et al. 2013), emotional awareness skills training (Uzun and Lok 2022), and cognitive based violence intervention for schizophrenia patients with a history of violence (Hsu and Ouyang 2021) were all reported to alleviate alexithymic characteristics.

### 3.7 Meta‐Analysis

#### 3.7.1 Unidimensional Alexithymia and Schizophrenia

The associations between unidimensional alexithymia and schizophrenia were assessed based on the mean scores of the TAS total scale. First, mean scores were pooled to calculate the overall TAS score in schizophrenia samples to compare it against the TAS's cut‐off point (with scores < 61 classified as an alexithymic profile). Second, a subgroup analysis was applied to the pooled mean score obtained in the first step comparing the mean TAS total score of paranoid vs non‐paranoid schizophrenia samples.

**Figure 2:**
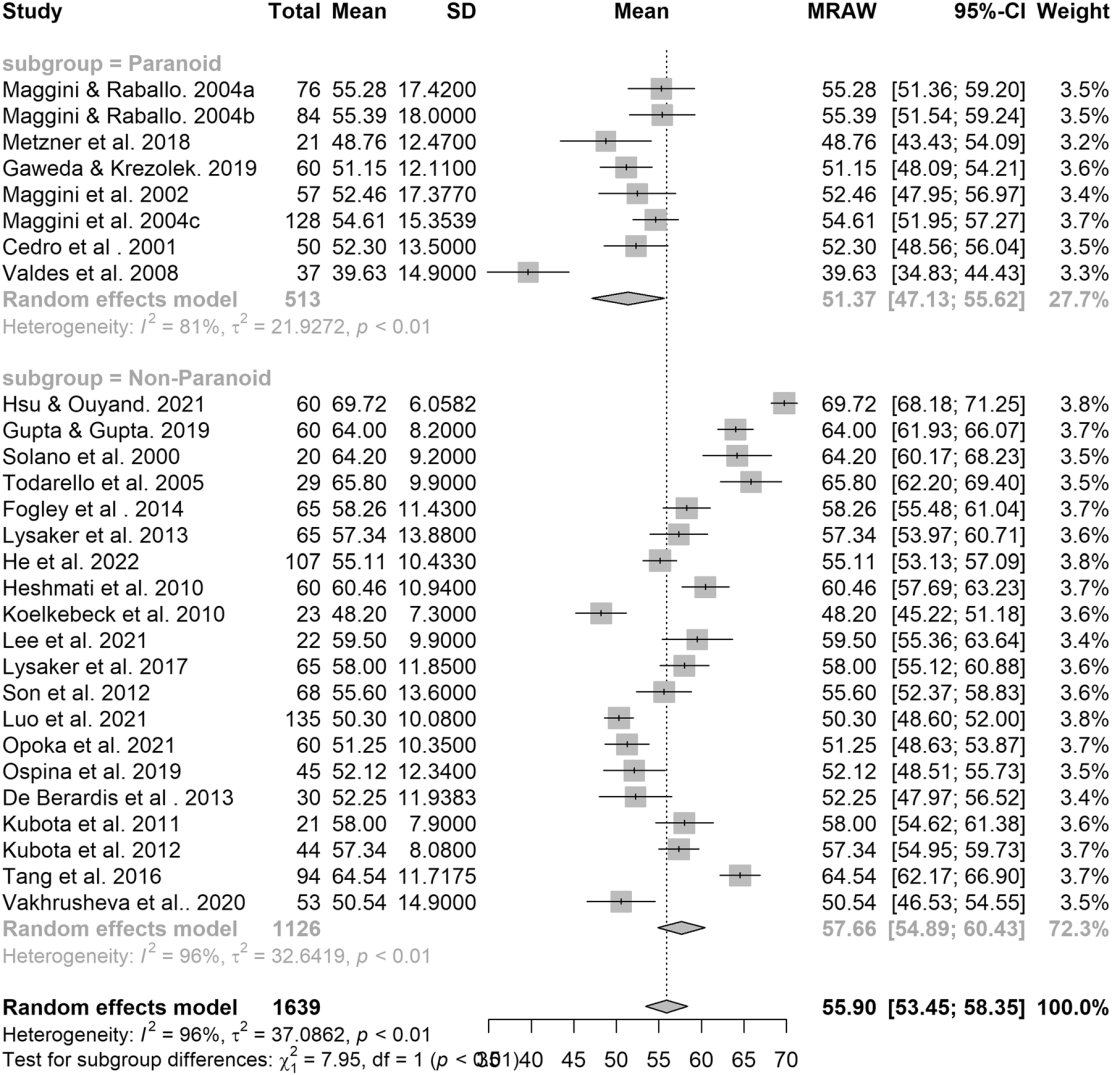
The difference of means between paranoid and non‐paranoid schizophrenia patients.

**Figure 3:**
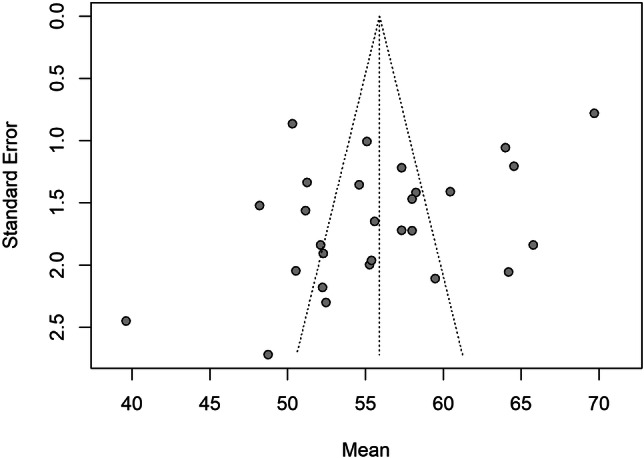
Funnel plot of the studies included in the meta‐analysis of means.

The restricted maximum‐likelihood estimator indicated substantial heterogeneity (*Q* = 562.30, *p* < 0.001) between the included 28 studies (*N* = 1639), with 96% of the variance in effect size due to between‐study variance. A random effects meta‐analysis of means was conducted to quantify the pooled mean of the TAS total score from 28 studies (*N* = 1639), which indicated a grand mean of 55.71 (95% CI = [53.07, 58.34]), below the TAS total score cut‐off point for classification of an alexithymic profile. The results were not moderated by age nor study quality. Subgroup analysis suggested non‐paranoid patients (*M* = 57.69, 95% CI = [54.61, 60.77]) reported significantly higher alexithymia scores compared to paranoid patients (*M* = 51.37, 95% CI = [47.12, 55.61]) (*p* < 0.01; see Figure 2). Funnel plot asymmetry suggested potential publication bias (see Figure 3). Seven studies were identified as outliers and removing the outliers rendered the difference between the groups to be nonsignificant.

### 3.8 Uni‐ and Multi‐Dimensional Alexithymia in Schizophrenia Compared to Nonclinical Samples

**Figure 4:**
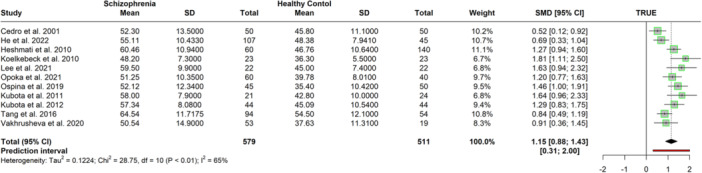
Meta‐Analysis of mean difference in the TAS total scores between schizophrenia patients and control groups after outliers were removed.

Next, we estimated the magnitude of associations between unidimensional alexithymia operationalized with the TAS total score, and multidimensional alexithymia operationalized with difficulties in identifying (TAS and BVAQ), describing (TAS and BVAQ), analyzing (BVAQ) feelings, emotionalizing (BVAQ), fantasizing (BVAQ), and externally oriented thinking (TAS) dimensions. Thirteen studies (*n* = 1372) reported comparisons between schizophrenia and nonclinical control samples. Case and control comparison could not be performed on the TAS total scores for paranoid patients because only two studies included a control group (Cedro et al. 2001; Valdés et al. 2008). Two studies were detected as outliers and influential cases (Luo et al. 2021; Valdés et al. 2008) and were removed from the analysis. The subsequent results based on 11 studies (N = 1090) suggested that non‐paranoid schizophrenia patients had significantly higher TAS total scores, with a positive large effect size (*g* = 1.15, 95% CI = [0.87, 1.43], *p* < 0.001) showing moderate heterogeneity (*I* ^*2*^ = 0.65) (see Figure 4 and Table 1). Differences were not moderated by age (β = −0.03, 95% CI = [−0.07, 0.01], *p* = 0.141), nor by study quality (β = 0.09, 95% CI = [−0.48, 0.68], *p* = 0.712). Eggers' test indicated no funnel plot asymmetry (β = 3.84, 95% CI = [0.41, 7.28], *p* = 0.055).

**Table 1:** Results of the meta‐analysis of standardized mean difference for TAS total.

	*g*	95%CI	*p*	95%PI	*I* ^2^	95%CI
Original analysis	1.05	0.77–1.32	*p* < 0.01	0.11–1.99	0.73	0.53–0.85
Sensitivity analysis^a^	1.15	0.87– 1.43	*p* < 0.001	0.31–1.99	0.65	0.34–0.82

^a^ Removed Studies: Luo et al. (2021) and Valdés et al. (2008).

**Figure 5:**
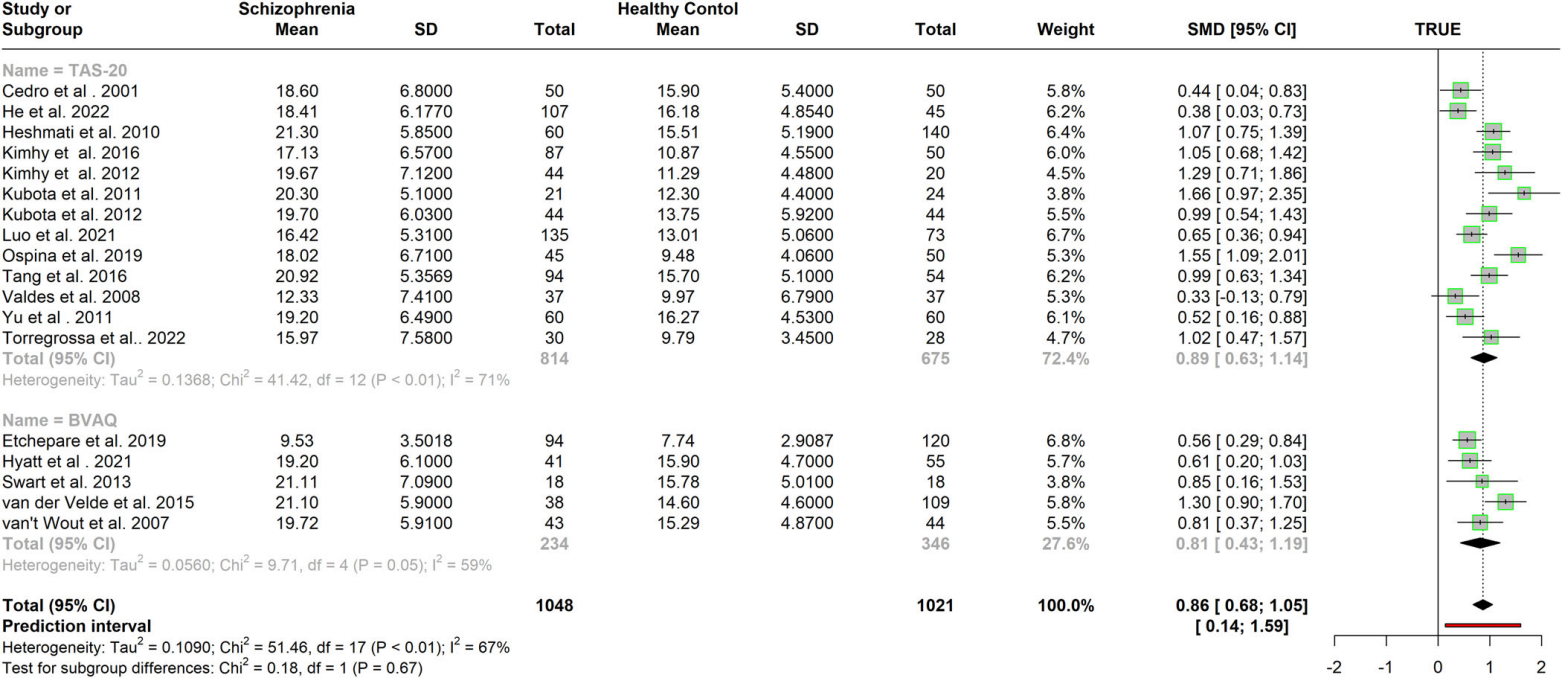
Meta‐Analysis of mean difference in difficulty in identifying feelings between schizophrenia patients and control groups after outliers were removed.

Further case‐control analyses were conducted on alexithymia dimensions. The TAS and the BVAQ scores were pooled for identifying and describing emotions dimensions and only TAS scores were pooled for externally oriented thinking. Scores obtained from additional dimensions assessed by the BVAQ involving fantasizing, emotionalizing, and analyzing were further compared between schizophrenia and control samples. The original analysis for identifying feelings involved 19 studies (*N* = 2129) and 1 study was detected as an outlier and influential case and removed from analysis (Kumar et al. 2018). Sensitivity analysis (*k* = 18; *n* = 2069) suggested a large positive effect associated with schizophrenia (*g* = 0.86, 95% CI = [0.67, 1.05], *p* < 0.001), with moderate heterogeneity (*I* ^*2*^ = 0.67) (see Table 2 for results of original and sensitivity analyses). Meta‐regression indicated study quality was a significant moderator, positively associated with the DIF scores (β = 0.25, 95% CI = 0.04, 0.46; *p* < 0.05), whilst age was not a moderator. Eggers' test did not suggest funnel plot asymmetry. Subgroup analysis was conducted on the revised model to compare the difficulties in identifying feelings scores obtained from the BVAQ and the TAS. The subgroup analysis presented in Figure 5 indicated a significant difference (*p* < 0.001) between the scores, in which the TAS (*k* = 13, *g* = 0.89, 95% CI = [0.63, 1.14]) generated higher scores on the difficulties in identifying feelings dimension than the BVAQ (*k* = 5, *g* = 0.81, 95% CI = [0.43, 1.19]).

**Table 2:** Results of the meta‐analysis of standardized mean difference for DIF.

	*g*	95%CI	*p*	95%PI	*I* ^2^	95%CI
Original analysis	0.94	0.70–1.18	*p* < 0.001	−0.06–1.94	0.76	0.62–0.84
Sensitivity analysis^a^	0.86	0.67–1.05	*p* < 0.001	0.14–1.59	0.67	0.46–0.80

^a^ Removed Study: Kumar et al. (2018).

**Figure 6:**
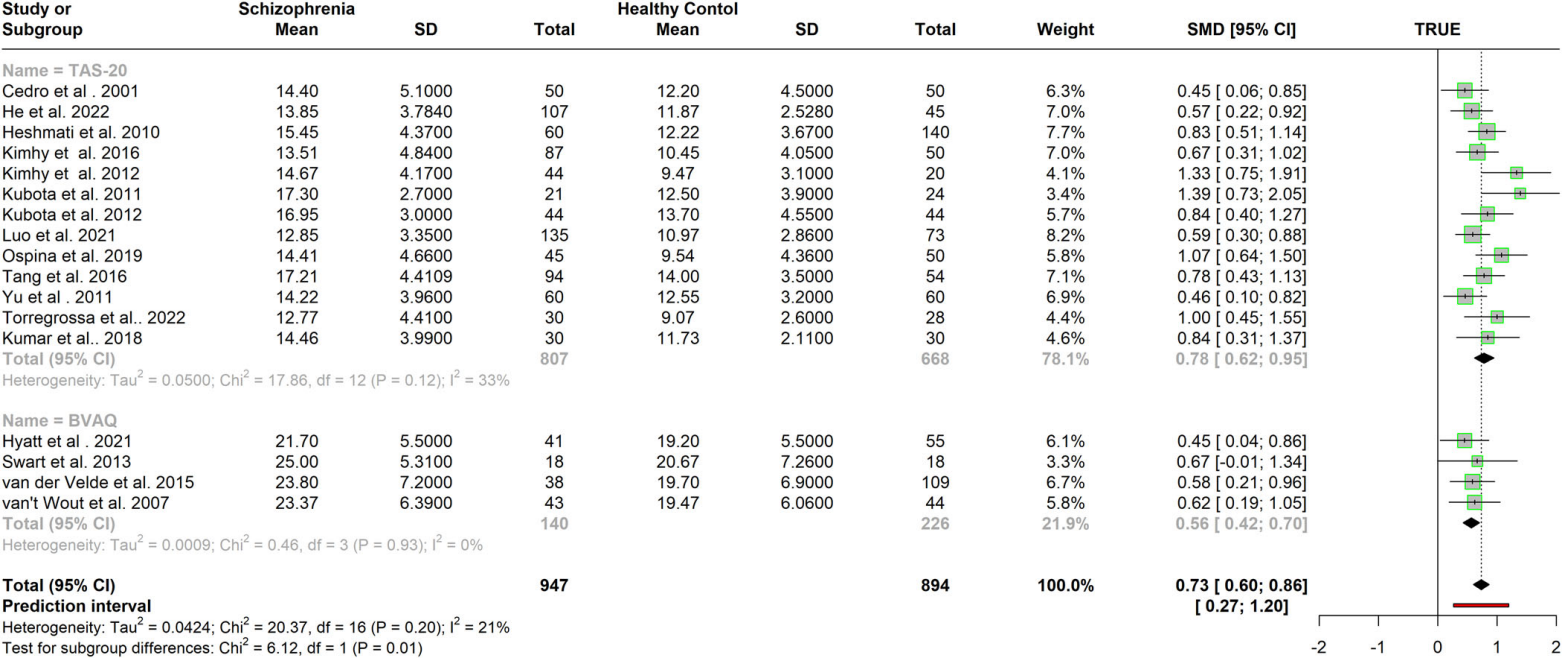
Meta‐Analysis of mean difference in difficulty in describing feelings between schizophrenia patients and control groups after outliers were removed.

Among 19 case‐control studies (*N* = 2129) that reported eligible data on difficulties in describing feelings, two studies were identified as outliers and influential cases (Etchepare et al. 2019; Valdés et al. 2008). Table 3 presents the results of the original and sensitivity analyses and Figure 6 provides the results grouped by alexithymia assessment (the TAS vs the BVAQ). The sensitivity analysis conducted after the exclusion of the influential cases suggested a medium effect positively associated with schizophrenia (*k* = 17; *n* = 1841; *g* = 0.73, 95% CI = [0.60, 0.86], *p* < 0.001), with low heterogeneity (*I* ^2^ = 0.21). Meta‐regression indicated age was a significant moderator, positively associated with the DDF scores (β = 0.01, 95% CI = 0.00, 0.03; *p* < 0.05). Study quality was not a moderator (β = 0.31, 95% CI = [−0.08, 0.70]; *p* = 0.11). Eggers' test suggested evidence of funnel plot asymmetry (intercept = 2.66, 95% CI = [0.58, 4.74], *p* < 0.05). Figure 6 shows the results of the subgroup analysis conducted on the revised model to compare the studies using the BVAQ and the TAS,. The subgroup analysis showed a significant difference (*p* < 0.001) indicating a higher mean score for the difficulties in describing feelings dimension as recorded by the TAS (*k* = 13, *g* = 0.78, 95% CI = [0.62, 0.95]) compare to the BVAQ (*k* = 4, *g* = 0.56, 95% CI = [0.42, 0.70]).

**Table 3:** Results of the meta‐analysis of standardized mean difference for DDF.

	*g*	95%CI	*p*	95%PI	*I* ^2^	95%CI
Original analysis	0.67	0.52–0.82	*p* < 0.001	0.09–1.25	0.50	0.15–0.71
Sensitivity analysis^a^	0.73	0.60–0.86	*p* < 0.001	0.27–1.19	0.21	0.0–0.56

^a^ Removed Studies: Etchepare et al. (2019) and Valdés et al. (2008).

**Figure 7:**
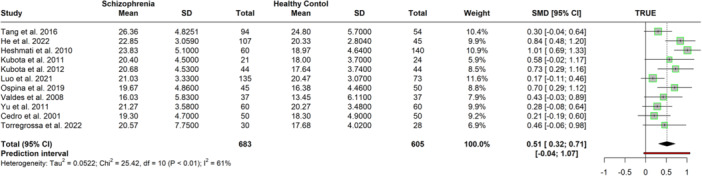
Meta‐Analysis of mean difference in externally oriented thinking between schizophrenia patients and control groups after outliers were removed.

Externally oriented thinking scores were reported by 12 studies (*N* = 1348). One study (Kumar et al. 2018) was found to be an outlier and an influential case, exclusion of which reduced heterogeneity from substantial to medium and revealed a medium effect positively associated with schizophrenia (*k* = 11; *n* = 1228, *g* = 0.56, 95% CI = [0.32, 0.71], *p* < 0.01, see Figure 7). Table 4 demonstrates the results of original and sensitivity analyses. Age (β = −0.01, 95% CI = −0.04, 0.03; *p* = 0.73) or study quality (β = 0.21, 95% CI = −0.06, 0.47; *p* = 0.12) did not moderate the tested model. Eggers' test did not indicate evidence for a funnel plot asymmetry (intercept = 0.96, 95% CI = [−4.02, 5.95], *p* = 0.71).

**Table 4:** Results of the meta‐analysis of standardized mean difference for EOT.

	*g*	95%CI	*p*	95%PI	*I* ^2^	95%CI
Original analysis	0.64	0.30–0.98	*p* < 0.01	−0.54–1.82	0.79	0.64–0.88
Sensitivity analysis^a^	0.51	0.32–0.71	*p* < 0.001	−0.04–1.07	0.61	0.24–0.80

^a^ Removed Study: Kumar et al. (2018).

Three dimensions distinct to BVAQ are analyzing feelings, emotionalizing, and fantasizing. Case‐control comparisons on these dimensions based on five studies (*N* = 580) indicated that healthy control samples do not differ from schizophrenia samples on any of the analyzing (β = 0.35, 95% CI = [−0.02, 0.72], *p* = 0.056), emotionalizing (β = 0.10, 95% CI = [−0.73, 0.94], *p* = 0.75), and fantasizing (β = 0.12, 95% CI = [−0.26, 0.49], *p* = 0.45) dimensions. However, two of these studies involved predominantly paranoid samples (Etchepare et al. 2019; van 't Wout et al. 2007).

### 3.9 Multidimensional Associations between Alexithymia and Schizophrenia

The results of the correlational meta‐analysis estimating the associations between schizophrenia, involving negative, positive, disorganized, and overall symptomatology, and alexithymia involving the TAS total score, DIF, DDF, and EOT are shown in Table 5. The pooled association between negative symptoms and the TAS total derived from 6 studies (*n* = 2998) was *r* = 0.30 (*p* < 0.05). Although the results were not moderated by study quality (β = −0.17, 95% CI = −0.43, 0.07; *p* = 0.12), Eggers’ test indicated presence of funnel plot asymmetry (intercept = −3.45, 95% CI = [−5.73, −1.18], *p* < 0.05). Studies reporting associations between difficulties identifying and describing feelings dimensions of alexithymia and negative symptoms involved community samples (*k* = 4, *n* = 3516). Only one study included a schizophrenia sample (Tang et al. 2016), which was excluded to specify the associations for subclinical negative symptoms and alexithymia. Moderate associations were found between negative symptoms and DDF (*r* = 0.46, *p* < 0.05) and DIF (*r* = 0.42, *p* < 0.05) dimensions of the TAS. The association between negative symptoms and EOT was nonsignificant (*r* = 0.22, *p* = 0.31). The only significant association involving alexithymia and positive symptoms was of identification (*k* = 5, *n* = 2926, *r* = 0.24, *p* < 0.05). The remaining associations between total alexithymia scores, DIF, DDF, and EOT dimensions and total PANSS scores and positive and disorganized symptoms were nonsignificant. The results are confined by the small number of studies, which also suggest nonsignificant but larger magnitude of associations observed between disorganized symptoms and general schizophrenia symptomatology and multidimensional alexithymia compared to positive symptoms.

**Table 5:** Results of the correlational meta‐analysis of the associations between schizophrenia symptom dimensions and unidimensional and multidimensional alexithymia.

	TAS total	DIF	DDF	EOT
**Negative symptoms**	0.30*	0.42*	0.39*	0.22
	[0.07–0.50]	[0.12–0.65]	[0.15–0.58]	[−0.45–0.73]
	*k* = 6, *n* = 2998	*k* = 4, *n* = 3516	*k* = 5, *n* = 3610	*k* = 3, *n* = 3384
**Positive symptoms**	0.11	0.24*	0.05	—
	[−0.16–0.38]	[0.02–0.44]	[−0.27– 0.36]	
	*k* = 6, *n* = 2909	*k* = 5, *n* = 2926	*k* = 4, *n* = 2926	
**Disorganized symptoms**	0.19	0.32	0.23	—
	[−0.62–0.81]	[−0.22–0.72]	[−0.19–0.59]	
	*k* = 3, *n* = 2827	*k* = 3, *n* = 2827	*k* = 3, *n* = 2827	
**PANSS total**	0.38	0.47	—	—
	[−0.1495–0.7428]	[−0.04–0.79]		
	*k* = 4, *n* = 2903	*k* = 3, *n* = 2808		

## 4 Discussion

The current systematic review and meta‐analysis sought to synthesize the evidence for the role of alexithymia as a unidimensional and multidimensional construct in schizophrenia by (1) assessing the severity of unidimensional alexithymia in individuals with schizophrenia, (2) examining sub‐group differences in unidimensional alexithymia within schizophrenia population based on the presence or absence of paranoia, (3) comparing nonclinical control groups with schizophrenia groups on both unidimensional and multidimensional alexithymia, and (4) exploring associations between multidimensional alexithymia and schizophrenia symptom domains.

Meta‐analysis identified that unidimensional alexithymia and schizophrenia were positively associated at a large magnitude of effect, confirming and extending previous meta‐analyses (O'Driscoll et al. 2014). In terms of multidimensional alexithymia, a large magnitude positive association was found between schizophrenia and DIF with moderate heterogeneity, while moderate positive associations were observed between schizophrenia and DDF with low heterogeneity and EOT with moderate heterogeneity. Contextualizing the meta‐analytic findings with our findings from our narrative synthesis suggests that alexithymia dimensions may relate differently to schizophrenia symptom dimensions. Although our findings are tentative, DDF may be of greater magnitude in psychosis than in affective and personality disorders (Caccamo et al. 2019), the difference of which was not observed in studies comparing the same populations based on the TAS total scores (Opoka et al. 2021; Ospina et al. 2019). Further, difficulties in identifying feelings were reported to be predominantly associated with positive symptoms (Maggini and Raballo 2004; Serper and Berenbaum 2008; Yu et al. 2011) and externally oriented thinking with depression and neurocognitive impairment (Fogley et al. 2014; Luo et al. 2021). These findings suggest the importance of investigating alexithymia multidimensionally, rather than as a unidimensional construct.

Moderate positive associations were found for the relationship between DIF and DDF and subclinical negative symptoms, whereas a small positive association was found between positive symptoms and DIF. Similar patterns of findings were observed in the narrative synthesis of studies exploring alexithymia in community samples, whereby DDF and DIF were associated with negative schizotypy (Larøi et al. 2008; Martin et al. 2016; Prince and Berenbaum 1993) and positive schizotypy was reported to be associated with DIF (Fung et al. 2017; van Rijn et al. 2011; Van 't Wout et al. 2004; Yang et al. 2020). The differential associations between alexithymia and symptom dimensions further support assessing alexithymia multidimensionally.

In terms of unidimensional alexithymia, the mean score obtained from schizophrenia samples indicated lack of evidence for an alexithymic profile in schizophrenia based on the cut‐off point of the TAS (Bagby et al. 1994) and that non‐paranoid schizophrenia patients reported more alexithymic impairment than paranoid schizophrenia patients. Only one study directly tested the difference between alexithymia scores in paranoid vs non‐paranoid schizophrenia, reporting the same pattern of results (Stanghellini and Ricca 1995). The finding regarding an elevated alexithymic profile in non‐paranoid schizophrenia compared to paranoid schizophrenia based on the unidimensional TAS score can be elaborated in relation to the severity of thought disorder or disorganization in non‐paranoid schizophrenia. Paranoid schizophrenia, as opposed to non‐paranoid schizophrenia, has been suggested to present with a relatively organized cognitive functioning (Sass and Feyaerts 2024). Disorganization as a schizotypy dimension showed stronger associations with affective dynamics compared to negative and positive schizotypy (Kemp et al. 2023) and identified as a predictor of daily experiences of negative affect and perceived uncontrollability of self‐experiences (Hernández et al. 2023). Similarly, the identification dimension of alexithymia, particularly, has been suggested to be associated with distress due to psychological disorder (Marchesi et al. 2014; Preece et al. 2024), which resonates with meta‐analytic findings on theory of mind impairments in schizophrenia reporting that difficulties in reasoning about others' intentions and in identifying others' mental states may escalate due to thought disorder severity in acute psychosis (Bora et al. 2009; Sprong et al. 2007). Further research is required to explore subgroup differences within schizophrenia populations due to heterogeneity of paranoid schizophrenia samples.

### 4.1 Limitations and Recommendations

Measurement issues should be noted in the interpretation of the results. Psychometric studies of the self‐report assessments of alexithymia indicated inconsistent evidence for the validity and reliability of the TAS and the BVAQ. Limitations of the TAS paradigm may involve issues with the scale's validity in discriminating alexithymia from psychological distress and psychometric reliability of the EOT dimension. In terms of validity, the TAS was suggested as an indicator of psychological distress due to symptom severity rather than representing the alexithymia construct (Marchesi et al. 2014) and the problematic discriminant validity may relate particularly to the DIF dimension, which psychometrically cross‐loaded onto alexithymia and general distress constructs (Preece et al. 2024).

Another psychometric issue pertains to reliability of the EOT dimension, yielding questionable metrics in psychometric studies of the TAS's translated versions (Bagby et al. 2020), which signals issues with generalizability due to cross‐cultural variance in EOT's psychometric properties. Importantly, meta‐analyses of alexithymia in various clinical populations suggested weak or nonsignificant associations between the EOT dimension and depression (Li et al. 2015), suicide (Hemming et al. 2019), self‐harm (Norman et al. 2020), substance use (Honkalampi et al. 2022), and eating disorders (Nowakowski et al. 2013), suggesting issues with the EOT dimension's construct validity. Consequently, our meta‐analytic finding of a moderate association between EOT and schizophrenia is limited by questionable psychometric support for the EOT subscale of the TAS.

The BVAQ, on the other hand, has been critiqued for its emotionalizing dimension as reflecting differences in physiological arousal rather than an alexithymic quality (Bagby et al. 2009). Importantly, a factor analytic study indicated that alexithymia as estimated by the DIF, DDF, and EOT dimensions was associated with elevated negative affective reactivity, not with a diminishment of affective reactivity as assumed in the BVAQ paradigm (Preece et al. 2017). Additionally, similar to the TAS, translated versions of the BVAQ yielded questionable internal reliability estimates (Sekely et al. 2018), which may indicate a problem with cultural invariance of the measures.

Our meta‐analysis indicated a significantly lower score obtained in the DIF and DDF dimensions of the BVAQ compared to the TAS in schizophrenia samples, and no statistically significant differences were observed between healthy control and schizophrenia samples on analyzing, emotionalizing, and fantasizing dimensions of the BVAQ. Results for the fantasizing dimension can be related to research on creativity suggesting an inverted‐U association for creativity across the psychosis continuum: positive schizotypy was positively associated with creativity (Acar and Sen 2013), while chronic schizophrenia and creativity was negatively associated (Acar et al. 2018). Conversely, the BVAQ's fantasizing dimension yielded nonsignificant estimates, which may indicate validity issues. However, the lack of a significant association may relate to the limited number of meta‐analyzed studies, which may prevent detection of small effect sizes. Future research may examine the differences between the TAS and the BVAQ in assessing alexithymia.

An important limitation of the literature on alexithymia in schizophrenia may be the inability to account for the fluctuations in alexithymia in relation to psychological distress. The static nature of cross‐sectional designs, which are prevalent in alexithymia research, prevents an understanding of the dynamic relationship between multidimensional alexithymia and psychological distress. Furthermore, the quality assessment showed that around 60% of the included studies did not control for distress‐related and other possible covariates of alexithymia which may inflate estimated effect sizes. Based on the systematic review, possible covariates are depression, anxiety, stress, schizophrenia symptom severity, neurocognitive deficits, age, and gender. These factors should be taken into account to isolate the influence of alexithymia on schizophrenia and psychosis for future research.

There is likely an issue regarding the quality appraisal tool not being sufficiently adapted for the specific requirements of this review in the context of ascertainment of exposure criterion. The studies collectively received null scores due to the use of self‐report assessments although this does not necessarily indicate bias in the quality assessment. Rather, it suggests that studies consistently met or failed to meet certain quality criteria to a similar extent. Another methodological issue is the lack of reporting of drop‐out rates and how missingness is handled, which were not reported in the included studies. Other limitations involved representativeness of participants to the population of interest, selection and definition of control groups.

A further area of research is investigating alexithymia's relation to psychosocial functioning. The results are mixed in terms of the relationship between alexithymia and psychosocial functioning. The majority of studies reported associations between alexithymia and psychosocial functioning (Kimhy et al. 2012, 2016; Luo et al. 2021; Seghers et al. 2011; Ustundag et al. 2020). However, it was also reported that when negative symptoms were controlled for, alexithymia's association to psychosocial functioning becomes nonsignificant (Todarello et al. 2005), which further highlights the need to include covariates into analytic models.

Issues such as to what extent an inability to express feelings may interact with diminished social relatedness (i.e., social anhedonia) and diminished verbal expressivity (i.e., alogia) also require further research. Clarification of the nature of these interactions may inform the development of interventions targeting functional/social functioning outcomes. Research on the associations between alexithymia and social functioning is limited and has produced mixed results: significant associations between alexithymia and social functioning become nonsignificant after negative symptoms were controlled for. To this end, panel or experience sampling designs can be used to model the synergistic interactions between alexithymia, psychological distress, and schizophrenia phenomenology and their relationship with social functioning. Future research can also benefit from investigating alexithymia in non‐paranoid and paranoid schizophrenia controlling for disorganization severity and elevated negative affect to understand the subgroup differences within schizophrenia population. Temporal modeling approaches (e.g., McNeish and Hamaker 2019) can be utilized to disentangle the associations between schizophrenia phenomenology and alexithymia accounting for negative affect or symptom severity. Furthermore, network modeling methods (e.g., Epskamp et al. 2018) can accommodate large sets of variables, which makes it particularly appropriate to apply considering the identified confounders of the association between schizophrenia and alexithymia.

## 5 Conclusion

The current systematic review identified robust associations between self‐reported alexithymia and schizophrenia diagnoses. In terms of theory and research, we suggest conceptualizing alexithymia multidimensionally. The multidimensional concept of alexithymia can be supported based on differential associations between dimensions of alexithymia and dimensions of schizophrenia symptomatology. For example, the correlational meta‐analysis indicated that difficulty in identifying feelings was associated with positive schizotypy, association of which was nonsignificant for the TAS total score. The reviewed research indicates that alexithymic impairments may be elevated due to schizophrenia symptom severity and that 60% of the included studies did not control for covariates such as symptom severity or psychological distress. This may lead to an overestimation of the effect sizes obtained in the meta‐analysis.

To address these limitations, future research should utilize network modeling to account for confounders and implement longitudinal designs to differentiate the influence of trait impairments in alexithymia from stress‐reactive fluctuations in it. Additionally, controlling for covariates such as symptom severity and psychological distress is crucial to obtaining more accurate effect sizes. Researchers should also explore the nuanced relationship between specific dimensions of alexithymia and particular schizophrenia symptoms, rather than relying solely on overall alexithymia scores. This approach will help clarify the complex interplay between alexithymia and schizophrenia, potentially leading to more targeted and effective clinical interventions.

## Supporting information

Supporting information.

## Data Availability

The data that support the findings of this study are available from the corresponding author upon reasonable request.
