# Supplementary material for: Alexithymia in Schizophrenia and Psychosis Vulnerability: A Systematic Review and Meta‐Analysis
Source: J Clin Psychol. 2025 Mar 19;81(6):410–24. doi: 10.1002/jclp.23788 (PMC12050105; doi:10.1002/jclp.23788)
Supplement: Supplementary file 1 — Supporting information. [file JCLP-81-410-s001.docx]

Supplementary Material

**Alexithymia in Schizophrenia and Psychosis Risk States: A systematic review and meta-analysis**

# Quality Assessment

The selection category involves four subsections:

1. *Adequacy of case definition* determined by the classification of diagnosis based on an external validation of diagnosis (e.g., DSM or ICD).
2. *Representativeness of the Cases* was evaluated based on the recruitment of participants through a hospital either as an inpatient or an outpatient. If the sample includes both in- and out-patients, the study receives a score of zero 0.
3. *Selection of controls* category assessed whether the study included a healthy control group.
4. *Definition of controls* should include the criteria for the control group indicating no history of psychosis.

The comparability category involves one subsection, to which 2 points can be assigned based on analyses of covariates such as demographic characteristics, comparison with other psychiatric cases, or other relevant psychological phenomena that may relate to how alexithymia may present itself.

The exposure category involves three subsections:

1. *Ascertainment of exposure* should be determined using a structured interview and none of the included studies used an interview measure to assess alexithymia.
2. *The method of ascertainment for cases and controls* in the exposure category was used to evaluate the assessment of alexithymia. If the study used the same measure to assess alexithymia in case and control samples, one-point is given.
3. *Non-response rate* subsection is to assess attrition and missingness of data, which was not reported in any of the included studies.

**2. Prisma 2020 Checklist**

| **Section and Topic** | **Item #** | **Checklist item** | **Location where item is reported** |
| --- | --- | --- | --- |
| **TITLE** | | |  |
| Title | 1 | Alexithymia in Schizophrenia and Psychosis Vulnerability: A systematic review and meta-analysis. | p. 1 |
| **ABSTRACT** | | |  |
| Abstract | 2 | See the PRISMA 2020 for Abstracts checklist. | p.3 |
| **INTRODUCTION** | | |  |
| Rationale | 3 | Describe the rationale for the review in the context of existing knowledge.  Alexithymia refers to a set of cognitive impairments involving identification and expression of and attitudes towards emotional experiences and an impoverishment of imaginal capacity. As a mechanism involved in affect regulation, alexithymia is implicated in negative affective reactivity, which was identified as a pathway to psychosis. Alexithymia has been most commonly assessed with self-report measures and psychometric support for the measurement models are inconsistent. A unidimensional conceptualization of alexithymia was traditionally posited and has been prevalently used in research. A meta-analysis based on the unidimensional alexithymia model suggested a large effect size of alexithymia positively associated with schizophrenia. A systematic review narratively synthesized research on multidimensional alexithymia and schizophrenia and suggested associations between difficulties in identifying and describing feelings and schizophrenia diagnosis. A comprehensive systematic review quantifying multidimensional alexithymia and symptom dimensions of schizophrenia is yet to be conducted. | p. 4-5 |
| Objectives | 4 | Provide an explicit statement of the objective(s) or question(s) the review addresses.  The current systematic review and meta-analysis aimed to synthesize the existing research by investigating the relationship between schizophrenia and alexithymia as a unidimensional and multidimensional research construct in the following domains: (1) assessment of the severity of unidimensional alexithymic impairment within schizophrenia samples, (2) comparison of the healthy control and schizophrenia groups on unidimensional and multidimensional alexithymia, (3) investigation of subgroup differences within schizophrenia population based on presence or absence of paranoia, and (4) exploration of the associations between alexithymia dimensions and schizophrenia symptom dimensions. | p. 5-6 |
| **METHODS** | | |  |
| Eligibility criteria | 5 | Specify the inclusion and exclusion criteria for the review and how studies were grouped for the syntheses.  Inclusion: We included research studies of psychotic experiences, encompassing subclinical, prodromal or clinical forms of negative, disorganized, and positive symptoms assessed in conjunction to a quantifiable measure of alexithymia.  Exclusion: We excluded qualitative studies, case studies, conference abstracts, review articles, meta-analyses, theses and dissertations. | p.7 |
| Information sources | 6 | Specify all databases, registers, websites, organisations, reference lists and other sources searched or consulted to identify studies. Specify the date when each source was last searched or consulted.  The search involved the following databases: PsycINFO, MEDLINE, Embase, and Web of Science. Google Scholar was searched to identify the articles that are not indexed in databases. The search was restricted to research articles, published after 1973 (when the concept of alexithymia was first articulated) and written or translated into English. | p. 7 |
| Search strategy | 7 | Present the full search strategies for all databases, registers and websites, including any filters and limits used.  Search terms combined psychosis OR psychoses OR psychotic OR schizo* OR paranoi* OR hallucinat* OR delusion* OR "ultra-high risk" OR prodrom* OR "at risk mental state" AND Alexithymia OR “affective symptom*”. | p.7 |
| Selection process | 8 | Specify the methods used to decide whether a study met the inclusion criteria of the review, including how many reviewers screened each record and each report retrieved, whether they worked independently, and if applicable, details of automation tools used in the process.  The online application Covidence (Covidence, 2022) was used for the screening process. Two researchers (EO, ZX) independently reviewed titles and abstracts of the first 3046 records. Consensus was reached via discussions over the rationale of the inclusion or exclusion decision to obtain a consensus across the screening and full-text review phases. | p.7 |
| Data collection process | 9 | Specify the methods used to collect data from reports, including how many reviewers collected data from each report, whether they worked independently, any processes for obtaining or confirming data from study investigators, and if applicable, details of automation tools used in the process.  Extracted data included sample sizes and sample characteristics, mean, standard deviations, and correlation coefficients where available, which were inputted to an Excel sheet. | p. 8 |
| Data items | 10a | List and define all outcomes for which data were sought. Specify whether all results that were compatible with each outcome domain in each study were sought (e.g. for all measures, time points, analyses), and if not, the methods used to decide which results to collect.  Assessments of alexithymia included the Toronto Alexithymia Scale (TAS) and the Bermond Vorst Alexithymia Questionnaire (BVAQ). Affect identification and affect description subscales of these measures are conceptually the same. The subscales specific to the BVAQ are analyzing affects, fantasizing, and emotionalizing, whereas externally oriented thinking is a TAS specific scale. The identification and description scales were analyzed and the scores generated by each scale were compared. The remaining subscales were separately analyzed. | p. 16-17 |
|  | 10b | List and define all other variables for which data were sought (e.g. participant and intervention characteristics, funding sources). Describe any assumptions made about any missing or unclear information.  Collected data included  Article: author, year  Study: sample characteristics involving country of origin, size, clinical vs general population, age, gender.  Participants: schizophrenia symptom levels, severity of psychometric psychosis vulnerability, and psychometric alexithymia indices | p.8  p.10 |
| Study risk of bias assessment | 11 | Specify the methods used to assess risk of bias in the included studies, including details of the tool(s) used, how many reviewers assessed each study and whether they worked independently, and if applicable, details of automation tools used in the process.  Quality assessment was undertaken using the Newcastle-Ottawa Assessment Scales, which is an 8-point scale assessing research quality based on three domains: selection, comparability, and exposure.  The selection category involves four subsections:   1. *Adequacy of case definition* determined by the classification of diagnosis based on an external validation of diagnosis (e.g., DSM or ICD). 2. *Representativeness of the Cases* was evaluated based on the recruitment of participants through a hospital either as an inpatient or an outpatient. If the sample includes both in- and out-patients, the study receives a score of zero 0. 3. *Selection of controls* category assessed whether the study included a healthy control group. 4. *Definition of controls* should include the criteria for the control group indicating no history of psychosis.   The comparability category involves one subsection, to which 2 points can be assigned based on analyses of covariates such as demographic characteristics, comparison with other psychiatric cases, or other relevant psychological phenomena that may relate to how alexithymia may present itself.  The exposure category involves three subsections:   1. *Ascertainment of exposure* should be determined using a structured interview and none of the included studies used an interview measure to assess alexithymia. 2. *The method of ascertainment for cases and controls* in the exposure category was used to evaluate the assessment of alexithymia. If the study used the same measure to assess alexithymia in case and control samples, one-point is given. 3. *Non-response rate* subsection is to assess attrition and missingness of data, which was not reported in any of the included studies. | p.9  supp. P.1 |
| Effect measures | 12 | Specify for each outcome the effect measure(s) (e.g. risk ratio, mean difference) used in the synthesis or presentation of results.  The meta-analytic method utilized inverse variance and Q-Profile to estimate confidence intervals of tau2 and tau. Random effects models were adjusted using the Hartung-Knapp method to calculate the confidence interval around the pooled effect.  Raw means and standard deviations were used as the parameter of central tendency for the meta-analysis of means. The restricted maximum-likelihood estimator was used to estimate heterogeneity variance (τ2) based on the meta-analysis of means. The Sidik-Jonkman estimator was to be used if the restricted maximum-likelihood estimator indicated substantial heterogeneity. Additionally, a subgroup analysis was conducted on the meta-analysis of means to compare samples of paranoid and non-paranoid schizophrenia patients.  Random effects meta-analyses of mean differences were conducted between schizophrenia and non-clinical samples to compare alexithymia levels. In these analyses, the Sidik-Jonkman estimator was used to assess heterogeneity and Hedges g to standardize the mean difference.  A meta-analysis of correlations was conducted to pool the correlation coefficients between alexithymia dimensions and schizophrenic symptom dimensions, and restricted maximum-likelihood estimators were used to calculate heterogeneity.  Results were assessed for outlying and influential studies. The “find.outliers” function in the {dmetar} package was used to detect extreme individual studies' effect sizes compared to the overall effect. Influence diagnostics were calculated based on leave-one-out method and were examined using Baujat, diagnostics, overall effect, and 𝐼2 heterogeneity plots. Results of the sensitivity analyses excluding the outlying and/or influential cases are reported.  Moderator analyses were conducted on the studies included in the sensitivity analyses. Age and study quality were identified as moderator variables. Publication bias was visually assessed using funnel plots and statistically assessed using Eggers’ test. | p.8-9 |
| Synthesis methods | 13a | Describe the processes used to decide which studies were eligible for each synthesis (e.g. tabulating the study intervention characteristics and comparing against the planned groups for each synthesis (item #5)).  The narrative synthesis was conducted on studies pooled based on 4 domains: unidimensional and multidimensional alexithymia in schizophrenia, unidimensional and multidimensional alexithymia in psychosis vulnerability, covariates of alexithymia, and alexithymia and social functioning.  Meta-analyzed data included:  Alexithymia indices  The TAS: total score, difficulties in identifying feelings, and externally oriented thinking  The BVAQ: 5-dimensional scores on identifying, describing, and analyzing feelings, fantasizing, and emotionalizing  Schizophrenia and psychosis vulnerability indices  Negative, positive, or disorganized symptom dimensions of schizophrenia, schizotypy or psychosis risk states  Extracted mean and standard deviation values and Pearsons correlation coefficients were pooled for the listed meta-analyses. | p.16:19 |
|  | 13b | Describe any methods required to prepare the data for presentation or synthesis, such as handling of missing summary statistics, or data conversions.  Studies that reported alexithymia scores of subgroups within the sample (e.g., female vs male) were aggregated using the following formula suggested by Cochrane Handbook for Systematic Reviews of Interventions:  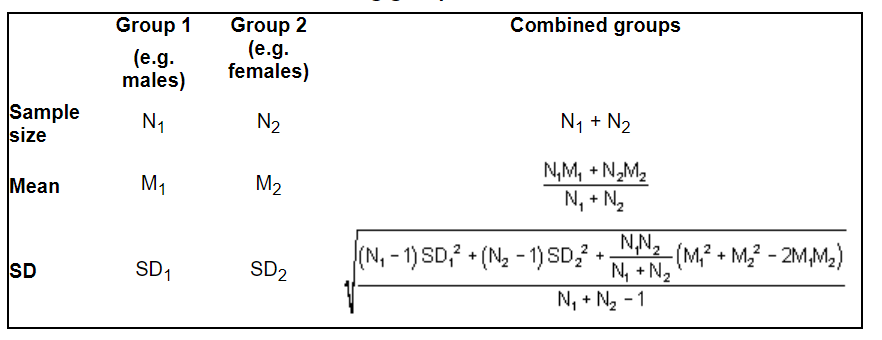 | N/A |
|  | 13c | Describe any methods used to tabulate or visually display results of individual studies and syntheses.  Forest and funnel plots were used to present the results of the meta-analyses. | p.9 |
|  | 13d | Describe any methods used to synthesize results and provide a rationale for the choice(s). If meta-analysis was performed, describe the model(s), method(s) to identify the presence and extent of statistical heterogeneity, and software package(s) used.  The R packages {meta} (Balduzzi et al., 2019), {dmetar} (Harrer et al., 2019), and {metafor} (Viechtbauer, 2010) were used to conduct the meta-analyses. The meta-analytic method utilized inverse variance and Q-Profile to estimate confidence intervals of tau2 and tau. Random effects models were adjusted using the Hartung-Knapp method to calculate the confidence interval around the pooled effect. The restricted maximum-likelihood estimator was used to estimate heterogeneity variance (τ2) based on the meta-analysis of means. The Sidik-Jonkman estimator was to be used if the restricted maximum-likelihood estimator indicated substantial heterogeneity. | p.8 |
|  | 13e | Describe any methods used to explore possible causes of heterogeneity among study results (e.g. subgroup analysis, meta-regression).  Subgroup analyses were conducted to compare paranoid and non-paranoid samples and effect sizes generated from different measurements of alexithymia.  Moderation analyses were conducted to control for the influence of study quality and mean age of the included samples. | p. 8-9 |
|  | 13f | Describe any sensitivity analyses conducted to assess robustness of the synthesized results.  Results were assessed for outlying and influential studies. The “find.outliers” function in the {dmetar} package was used to detect extreme individual studies' effect sizes compared to the overall effect. Influence diagnostics were calculated based on leave-one-out method and were examined using Baujat, diagnostics, overall effect, and 𝐼2 heterogeneity plots. Results of the sensitivity analyses excluding the outlying and/or influential cases are reported. | p.9 |
| Reporting bias assessment | 14 | Describe any methods used to assess risk of bias due to missing results in a synthesis (arising from reporting biases).  Publication bias was visually assessed using funnel plots and statistically assessed using Eggers’ test. | P.9 |
| Certainty assessment | 15 | Describe any methods used to assess certainty (or confidence) in the body of evidence for an outcome.  Not applicable | N/A |
| **RESULTS** | | |  |
| Study selection | 16a | Describe the results of the search and selection process, from the number of records identified in the search to the number of studies included in the review, ideally using a flow diagram  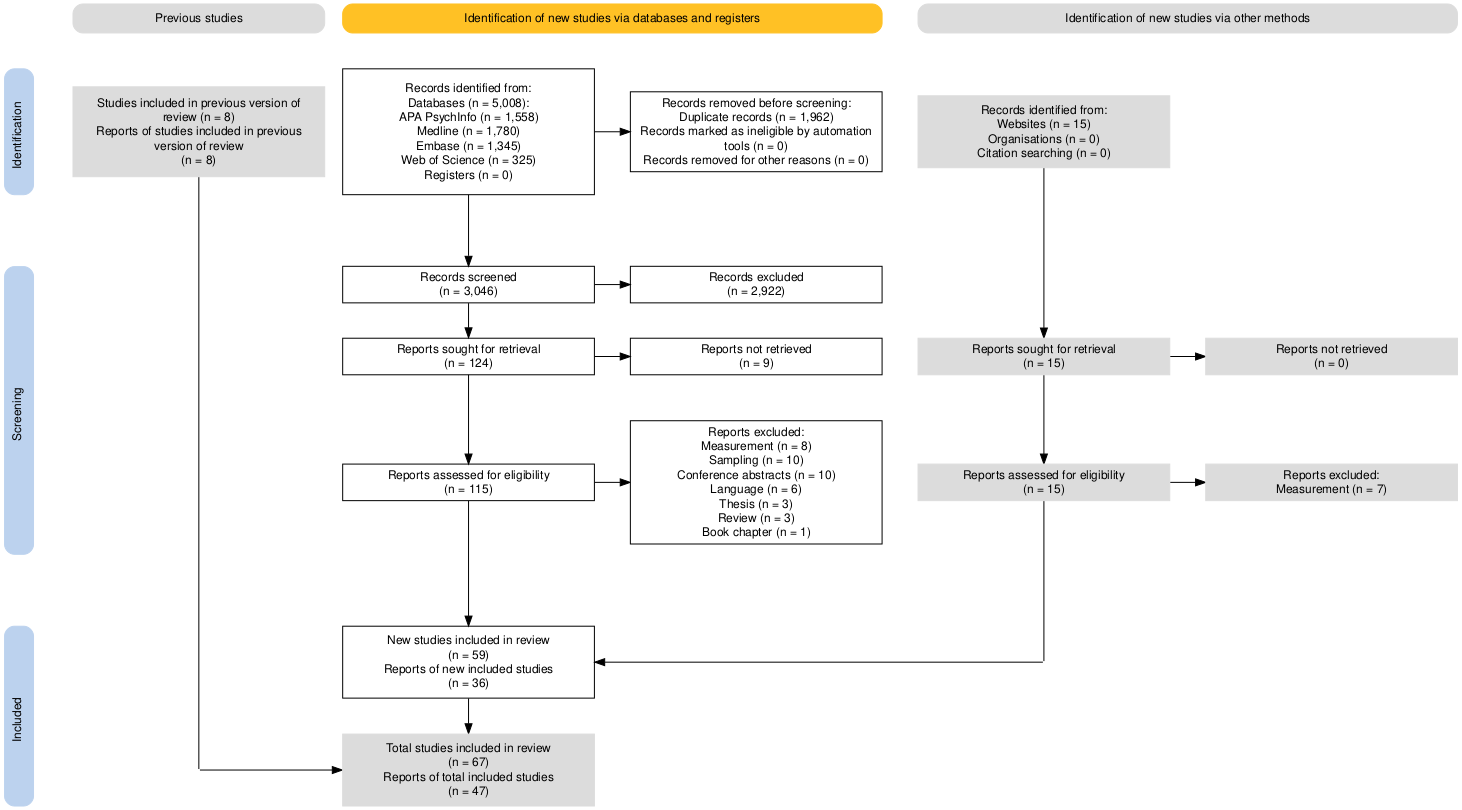  **Figure 1.** PRISMA flow diagram of the review process. | N/A |
|  | 16b | Cite studies that might appear to meet the inclusion criteria, but which were excluded, and explain why they were excluded.   \| **Study** \| **Exclusion Reason** \| \| --- \| --- \| \| (Boden & Berenbaum, 2012) \| Schizophrenia or psychotic experiences were not measured \| \| (Cernis et al., 2022) \| Alexithymia was not assessed \| \| (D’Antonio et al., 2015) \| Idiosyncratic scaling of the TAS based on 5 items. \| \| (Kolavarambath et al., 2020) \| Alexithymia was not assessed \| \| **References**  Boden, M., & Berenbaum, H. (2012). Facets of emotional clarity and suspiciousness. *PERSONALITY AND INDIVIDUAL DIFFERENCES*, *53*(4), 426–430. https://doi.org/10.1016/j.paid.2012.04.010  Cernis, E., Molodynski, A., Ehlers, A., & Freeman, D. (2022). Dissociation in patients with non-affective psychosis: Prevalence, symptom associations, and maintenance factors. *SCHIZOPHRENIA RESEARCH*, *239*, 11–18. https://doi.org/10.1016/j.schres.2021.11.008  D’Antonio, E., Kahn, J., McKelvey, J., Berenbaum, H., & Serper, M. (2015). Emotional awareness and delusions in schizophrenia and schizoaffective disorder. *COMPREHENSIVE PSYCHIATRY*, *57*, 106–111. https://doi.org/10.1016/j.comppsych.2014.10.006  Kolavarambath, R., Sudhir, P. M., Prathyusha, P. V., & Thirthalli, J. (2020). Emotion Recognition, Emotion Awareness, Metacognition, and Social Functioning in Persons with Schizophrenia. *Indian Journal of Psychological Medicine*, *42*(2), 147–154. https://doi.org/10.4103/IJPSYM.IJPSYM_149_19 \| \| | N/A |
| Study characteristics | 17 | Cite each included study and present its characteristics.  Refer to the Supplementary Materials |  |
| Risk of bias in studies | 18 | Present assessments of risk of bias for each included study.  Refer to the Supplementary Materials |  |
| Results of individual studies | 19 | For all outcomes, present, for each study: (a) summary statistics for each group (where appropriate) and (b) an effect estimate and its precision (e.g. confidence/credible interval), ideally using structured tables or plots.  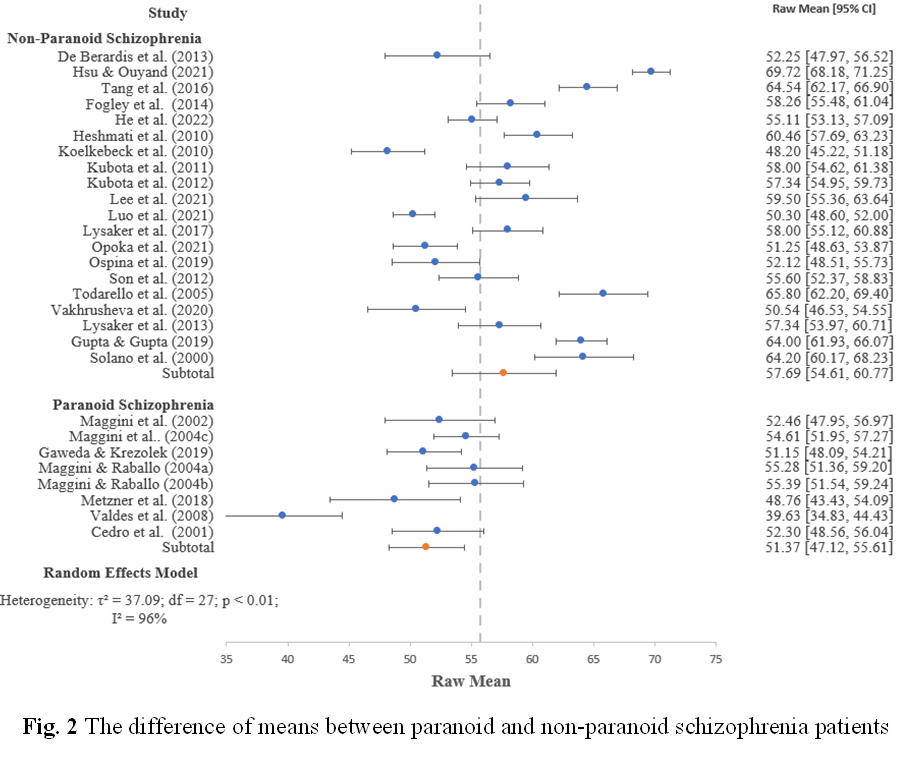    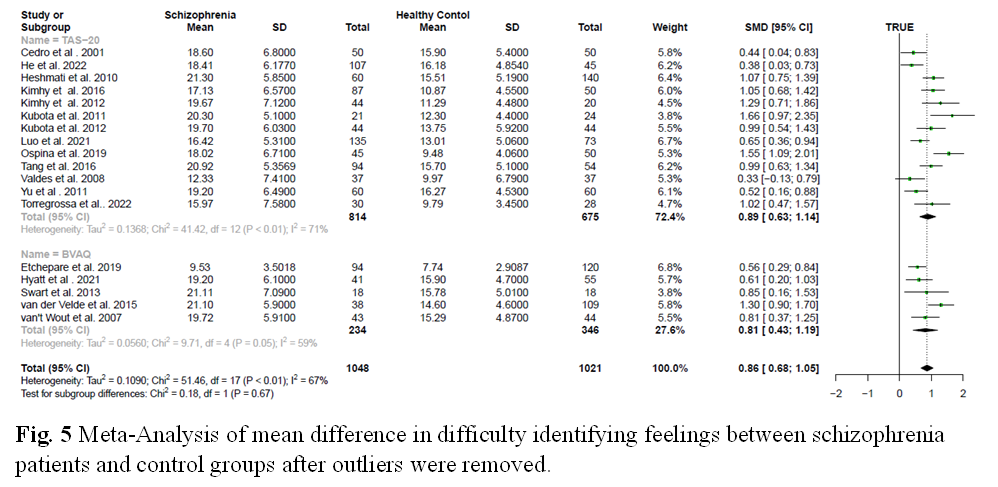  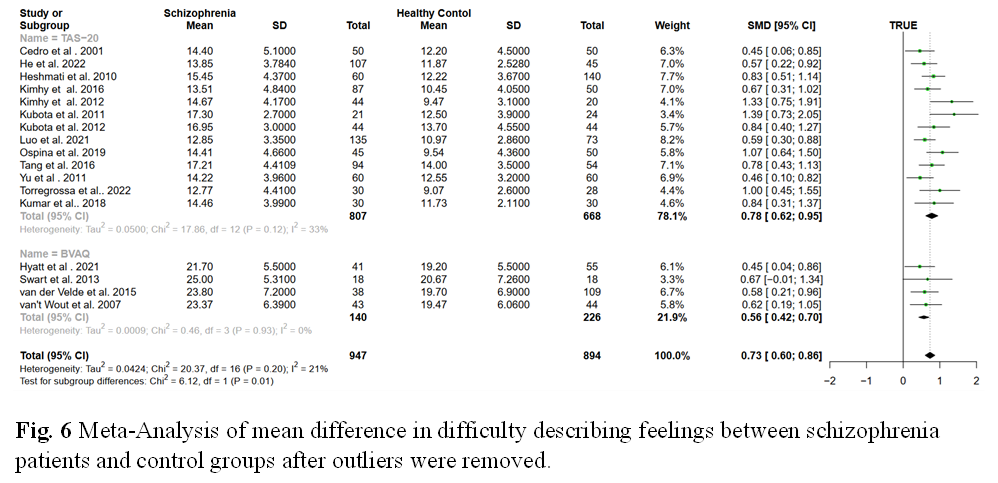  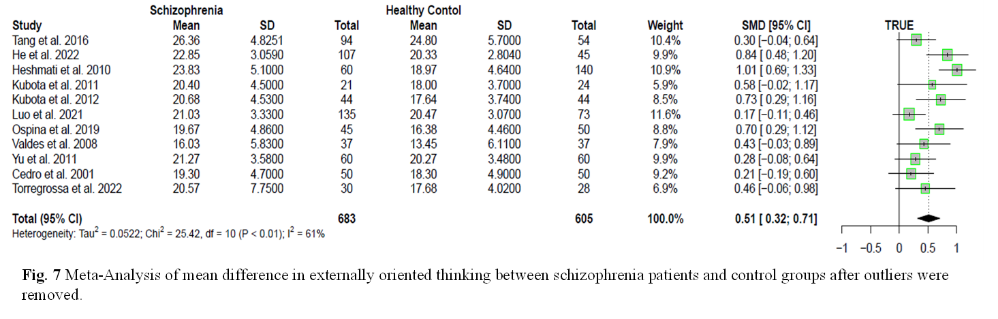 | N/A |
| Results of syntheses | 20a | For each synthesis, briefly summarise the characteristics and risk of bias among contributing studies.  The difference of means meta-analysis of the TAS total score Among the 28 studies (N = 1639), five studies were rated poor quality (Maggini & Raballo. 2004a, Maggini & Raballo. 2004b, Metzner et al. 2018, Hsu & Ouyand. 2021, Gupta & Gupta. 2019). Nine were rated good quality and 14 studies received fair quality scores.  Meta-analysis of the mean difference in the TAS total score involved 13 studies, four of which were fair quality (Cedro et al., 2001; He et al., 2022; Heshmati et al., 2010; Koelkebeck et al., 2010) and nine of which were good quality studies (Kubota et al., 2011; Kubota et al., 2012; Lee et al., 2021; Luo et al., 2021; Opoka et al., 2021; Ospina et al., 2019; Tang et al., 2016; Valdes et al., 2008; Vakhrusheva et al. 2020).  Meta-analysis of the mean difference in the difficulties in identifying feelings and difficulties describing feelings scores were obtained from the same 15 studies (n = 1048). Five studies received a poor quality rating (Cedro et al., 2001; He et al., 2022; Heshmati et al., 2010; van't Wout et al., 2007; Yu et al., 2011) and 13 studies received a fair quality rating (Etchepare et al. 2019; Hyatt et al . 2021; Kimhy et al. 2016; Kimhy et al. 2012; Kubota et al. 2011; Kubota et al. 2012; Luo et al. 2021; Ospina et al. 2019; Swart et al. 2013; Tang et al. 2016; Torregrossa et al.. 2022; Valdes et al. 2008; van der Velde et al. 2015). None of the studies received a good quality rating.  Meta-analysis of the mean difference in the externally oriented thinking scores were obtained from 12 studies (n = 713). None of the studies received a poor study quality rating. Four studies received a fair quality rating (Cedro et al. 2001; He et al. 2022; Heshmati et al. 2010; Yu et al. 2011) and the remaining studies were good quality. | Sup. |
|  | 20b | Present results of all statistical syntheses conducted. If meta-analysis was done, present for each the summary estimate and its precision (e.g. confidence/credible interval) and measures of statistical heterogeneity. If comparing groups, describe the direction of the effect.  The meta-analysis of means estimated on the TAS total scores included 28 studies (N = 1639). The analysis indicated a grand mean of 55.71 (95% CI = [53.07, 58.34]), with substantial heterogeineity (I^2^ = 96%). A Subgroup analysis found that that non-paranoid patients (M = 57.69, 95% CI = [54.61, 60.77]) reported significantly higher alexithymia scores compared to paranoid patients (M = 51.37, 95% CI = [47.12, 55.61]) (p < 0.01). Seven studies were identified as outliers and influential cases (Hsu & Ouyand. 2021; Gupta & Gupta. 2019; Solano et al. 2000; Todarello et al. 2005; Valdes et al. 2008; Luo et al. 2021; Tang et al. 2016) and removing these from the analysis generated a non-significant result.  Results of the meta-analysis of case-control comparisons are as follows;   1. non-paranoid schizophrenia patients had significantly higher TAS total scores, with a positive large effect size (g = 1.15, 95% CI = [0.87, 1.43], p < 0.001) showing moderate heterogeneity (I2 = 0.65). Differences were not moderated by age (β = -0.03, 95% CI = [-0.07, 0.01], p = 0.141), nor by study quality (β = 0.09, 95% CI = [-0.48, 0.68], p = 0.712). Eggers’ test indicated no funnel plot asymmetry (β = 3,84, 95% CI = [0.41, 7.28], p = 0.055). 2. The original analysis for identifying feelings involved 19 studies (N = 2129) and 1 study was detected as an outlier and influential case and removed from analysis (Kumar et al., 2018). Sensitivity analysis (k = 18; n = 2069) suggested a large positive effect associated with schizophrenia (g = 0.86, 95% CI = [0.67, 1.05], p < 0.001), with moderate heterogeneity (I2 = 0.67) (see Table 2 for the results of original and sensitivity analyses). Meta-regression analyses indicated study quality was a significant moderator (β = 0.25, 95% CI = 0.04, 0.46; p < 0.05), whilst age was not a moderator. Eggers’ test did not suggest funnel plot asymmetry. 3. Among 19 case-control studies (N = 2129) that reported eligible data on difficulties in describing feelings, 2 studies were identified as outliers and influential cases (Etchepare et al., 2019; Valdes et al., 2008). The sensitivity analysis conducted after the exclusion of the influential cases suggested a medium effect positively associated with schizophrenia (k = 17; n = 1841; g = 0.73, 95% CI = [0.60, 0.86], p < 0.001), with low heterogeneity (I2 = 0.21) (k = 17; n = 1841; Table 3). Meta-regression indicated age was a significant moderator (β = 0.01, 95% CI = 0.00, 0.03; p < 0.05), but study quality was not a moderator (β = 0.31, 95% CI = [-0.08, 0.70]; p = 0.11). Eggers’ test suggested evidence of funnel plot asymmetry (intercept = 2.66, 95% CI = [0.58, 4.74], p < 0.05). 4. Externally oriented thinking scores were reported by 12 studies (N = 1348). One study (Kumar et al., 2018) was found to be an outlier and an influential case, exclusion of which reduced heterogeneity from substantial to medium and revealed a medium effect positively associated with schizophrenia (k = 11; n = 1228, g = 0.56, 95% CI = [0.32, 0.71], p < 0.01). Age (β = -0.01, 95% CI = -0.04, 0.03; p = 0.73) or study quality (β = 0.21, 95% CI = -0.06, 0.47; p = 0.12) did not moderate the tested model. Eggers’ test did not indicate evidence for a funnel plot asymmetry (intercept = 0.96, 95% CI = [-4.02, 5.95], p = 0.71). 5. Three dimensions distinct to BVAQ are analyzing feelings, emotionalizing, and fantasizing. Case-control comparisons on these dimensions based on five studies (N = 580) indicated that healthy control samples do not differ from schizophrenia samples on any of the analyzing (β = 0.35, 95% CI = [-0.02, 0.72], p = .056), emotionalizing (β = 0.10, 95% CI = [-0.73, 0.94], p = .75), and fantasizing (β = 0.12, 95% CI = [-0.2664, 0.4976], p = .45) dimensions. However, two of these studies involved predominantly paranoid participants (Etchepare et al., 2019; van ’t Wout et al., 2007).   The correlational meta-analysis estimated the associations between schizophrenia, involving negative, positive, disorganized, and overall symptomatology, and alexithymia involving the TAS total score, DIF, DDF, and EOT.  The pooled association between negative symptoms and the TAS total derived from 6 studies (n = 2998) was 𝑟 = 0.30 (p < 0.05). Although the results were not moderated by study quality (β = -0.17, 95% CI = -0.43, 0.07; p = 0.12), Eggers' test indicated presence of funnel plot asymmetry (intercept = -3.45, 95% CI = [-5.73, -1.18], p < 0.05).  Studies reporting associations between difficulties in identifying and describing feelings dimensions of alexithymia and negative symptoms involved community samples (k = 4, n = 3516). Only one study included a schizophrenia sample (Tang et al., 2016), which was excluded to specify the associations for subclinical negative symptoms and alexithymia. Moderate associations were found between negative symptoms and difficulties describing feelings (𝑟 = 0.46, p < 0.05) and difficulties identifying feelings (𝑟 = 0.42, p < 0.05) dimensions of the TAS.  The association between negative symptoms and externally oriented thinking was non-significant (EOT; 𝑟 = 0.22, p = 0.31). The only significant association involving alexithymia and positive symptoms was of identification (k = 5, n = 2926, 𝑟 = 0.24, p < 0.05).  The remaining associations between total alexithymia scores, DIF, DDF, and EOT dimensions and total PANSS scores and positive and disorganized symptoms were non-significant (see the table below)   \| Results of the correlational meta-analysis of the associations between schizophrenia symptom dimensions and unidimensional and multidimensional alexithymia \| \| \| \| \| \| --- \| --- \| --- \| --- \| --- \| \|  \| TAS total \| DIF \| DDF \| EOT \| \| Negative  symptoms \| 0.30*  [0.07 - 0.50]  k = 6, n = 2998 \| 0.42*  [0.12 - 0.65]  k = 4, n = 3516 \| 0.39*  [0.15 - 0.58]  k = 5, n = 3610 \| 0.22  [-0.45 - 0.73]  k = 3, n = 3384 \| \| Positive  symptoms \| 0.11  [-0.16 - 0.38]  k = 6, n = 2909 \| 0.24*  [0.02 - 0.44]  k = 5, n = 2926 \| 0.05  [-0.27 - 0.36]  k = 4, n = 2926 \| - \| \| Disorganized  symptoms \| 0.19  [-0.62 - 0.81]  k = 3, n = 2827 \| 0.32  [-0.22 - 0.72]  k = 3, n = 2827 \| 0.23  [-0.19 - 0.59]  k = 3, n = 2827 \| - \| \| PANSS  total \| 0.38  [-0.1495 - 0.7428]  k = 4, n = 2903 \| 0.47  [-0.04 - 0.79]  k = 3, n = 2808 \| - \| - \| | p. 16-20 |
|  | 20c | Present results of all investigations of possible causes of heterogeneity among study results.  Refer to 20b. | N/A |
|  | 20d | Present results of all sensitivity analyses conducted to assess the robustness of the synthesized results.   \| Table 1 Results of the meta-analysis of standardized mean difference for TAS total \| \| \| \| \| \| \| \| --- \| --- \| --- \| --- \| --- \| --- \| --- \| \|  \| *g* \| 95%CI \| *p* \| 95%PI \| *I*^2^ \| 95%CI \| \| Original Analysis \| 1.05 \| 0.77-1.32 \| p < 0.01 \| 0.11-1.99 \| 0.73 \| 0.53-0.85 \| \| Sensitivity Analysis^1^ \| 1.15 \| 0.87- 1.43 \| p < 0.001 \| 0.31-1.99 \| 0.65 \| 0.34-0.82 \| \| ^1^Removed Studies: Luo et al. (2021) and Valdes et al. (2008) \| \| \| \| \| \| \|  \| Table 2 Results of the meta-analysis of standardized mean difference for DIF \| \| \| \| \| \| \| \| --- \| --- \| --- \| --- \| --- \| --- \| --- \| \|  \| *g* \| 95%CI \| *p* \| 95%PI \| *I*^2^ \| 95%CI \| \| Original Analysis \| 0.94 \| 0.70-1.18 \| p < 0.001 \| -0.06-1.94 \| 0.76 \| 0.62-0.84 \| \| Sensitivity Analysis^1^ \| 0.86 \| 0.67-1.05 \| p < 0.001 \| 0.14-1.59 \| 0.67 \| 0.46-0.80 \| \| ^1^Removed Study: Kumar et al. (2018) \| \| \| \| \| \| \|  \| Table 3 Results of the meta-analysis of standardized mean difference for DDF \| \| \| \| \| \| \| \| --- \| --- \| --- \| --- \| --- \| --- \| --- \| \|  \| *g* \| 95%CI \| *p* \| 95%PI \| *I*^2^ \| 95%CI \| \| Original Analysis \| 0.67 \| 0.52-0.82 \| p < 0.001 \| 0.09-1.25 \| 0.50 \| 0.15-0.71 \| \| Sensitivity Analysis^1^ \| 0.73 \| 0.60-0.86 \| p < 0.001 \| 0.27-1.19 \| 0.21 \| 0.0-0.56 \| \| ^1^Removed Studies: Etchepare et al. (2019) and Valdes et al. (2008) \| \| \| \| \| \| \| \|  \| \| \| \| \| \| \| \| Table 4 Results of the meta-analysis of standardized mean difference for EOT \| \| \| \| \| \| \| \|  \| *g* \| 95%CI \| *p* \| 95%PI \| *I*^2^ \| 95%CI \| \| Original Analysis \| 0.64 \| 0.30-0.98 \| p < 0.01 \| -0.54-1.82 \| 0.79 \| 0.64-0.88 \| \| Sensitivity Analysis^1^ \| 0.51 \| 0.32-0.71 \| p < 0.001 \| -0.04-1.07 \| 0.61 \| 0.24-0.80 \| \| ^1^Removed Study: Kumar et al. (2018) \| \| \| \| \| \| \| | N/A |
| Reporting biases | 21 | Present assessments of risk of bias due to missing results (arising from reporting biases) for each synthesis assessed.  Only the meta-analysis of means results indicated publication bias based on the below funnel plot. A publication bias was not suggested in the remaining meta-analyses 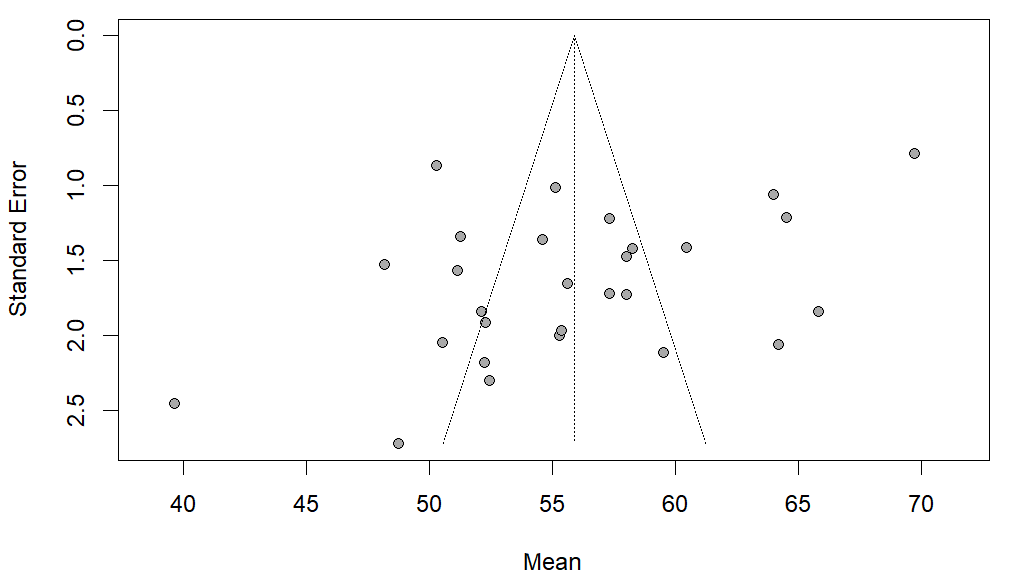 Funnel plot of the studies included in the meta-analysis of means | N/A |
| Certainty of evidence | 22 | Present assessments of certainty (or confidence) in the body of evidence for each outcome assessed.  Not applicable | N/A |
| **DISCUSSION** | | |  |
| Discussion | 23a | Provide a general interpretation of the results in the context of other evidence.  The meta-analysis identified that unidimensional alexithymia was positively associated with schizophrenia, at a large magnitude of effect, consistent with the the previous meta-analysis (O’Driscoll et al., 2014). Results of the meta-analysis support the findings of a previous systematic review evaluating the role of difficulties identifying and describing feeling dimensions in psychosis (Lawlor et al., 2020). In terms of the multidimensional alexithymia, a large magnitude positive association was found between schizophrenia and difficulties in identifying feelings with moderate heterogeneity, while moderate positive associations were observed between schizophrenia and difficulties in describing feelings with low heterogeneity and externally oriented thinking with moderate heterogeneity.  Alexithymia may be a set of cognitive impairments associated with schizophrenia phenomenology | p. 20-21 |
|  | 23b | Discuss any limitations of the evidence included in the review.  Inconsistent psychometric support for the self-report assessments of alexithymia (TAS and BVAQ), which were used among the included studies, and statistically significant differences between the TAS and the BVAQ in estimating alexithymia severity require caution in interpreting the results.  Lack of inclusion of covariates of alexithymia in analytic models is a further issue reducing reliability of the results. The quality assessment showed that around 60% of the included studies did not control for possible covariates of alexithymia. Based on the systematic review, possible covariates are depression, anxiety, stress, schizophrenia symptom severity, neurocognitive deficits, age, and gender. These factors should be taken into account to isolate the influence of alexithymia on schizophrenia and psychosis for future research. | p.23-26 |
|  | 23c | Discuss any limitations of the review processes used.  The review process was ensured the accuracy of the extracted data. Each step of the review process involving title/abstract screening, full-text review, data extraction, and quality assessment were independently undertaken and cross-validated by EO and ZX. | p. 7 |
|  | 23d | Discuss implications of the results for practice, policy, and future research.  The literature is premature to suggest clinical intervention targets. However, the systematic review has theory and research implications. In terms of theory, alexithymia can be contextualized within the higher order theoretical construct of mentalization and can be conceptualized as an impaired mentalizing process of identifying, reflecting on, and describing emotions. Alexithymia conceptualized as a multidimensional construct may have research utility to evaluate the dynamic interactions between affective dysregulation and psychosis. The literature can benefit from designing longitudinal studies using mixed methods approaches to gain a methodologically sound understanding of the role of multidimensional alexithymia in schizophrenia and psychosis. | p. 26-27 |
| **OTHER INFORMATION** | | |  |
| Registration and protocol | 24a | Provide registration information for the review, including register name and registration number, or state that the review was not registered.  The review protocol was registered in PROSPERO (ID number CRD42022327375). | p. 6 |
|  | 24b | Indicate where the review protocol can be accessed, or state that a protocol was not prepared. | p.6 |
|  | 24c | Describe and explain any amendments to information provided at registration or in the protocol.  The deviations from the registered protocol include revisions to the research question and the inclusion/exclusion criteria. The original research question, “What is the strength of the association between alexithymia and psychotic phenomenology, symptoms, and functioning?” was further elaborated in the four-domain formulation of the research aims. Additionally, the exclusion criteria were extended to involve conference abstracts, review articles, meta-analyses, theses and dissertations beyond qualitative and case studies. | p.6 |
| Support | 25 | Describe sources of financial or non-financial support for the review, and the role of the funders or sponsors in the review.  Not applicable | N/A |
| Competing interests | 26 | Declare any competing interests of review authors.  No competing interest to declare | N/A |
| Availability of data, code and other materials | 27 | Report which of the following are publicly available and where they can be found: template data collection forms; data extracted from included studies; data used for all analyses; analytic code; any other materials used in the review.  Data and the R code used for analyses can be made available upon request from the first author (EO). | N/A |

# Table S1

| **Table S1**  Summary of studies included in systematic review | | | | | | | | | | |
| --- | --- | --- | --- | --- | --- | --- | --- | --- | --- | --- |
| Year | Author | Sample  Type | N-Case | | N-Control | Gender-case  (female) | Alexithymia Measure | Diagnostic tool | TAS Total Mean (SD) | Quality |
| 2022 | He et al. | Non-paranoid | 107 | 45 | | 51% | TAS-20 | DSM-5 | 55.11  (10.43) | 5 |
| 2022 | Uzun & Lok | Non-paranoid | 22 | 22 | | 50% | TAS-20 | ICD-10 | - | 3 |
| 2022 | Torregrossa et al. | Non-paranoid | 30 | 28 | | 47% | TAS-20 | DSM-5 | - | 7 |
| 2021 | Hsu & Ouyand | Non-paranoid | 60 | - | | 45% | TAS-20 | ICD-10 | 69.72  (6.06) | 2 |
| 2021 | Hyatt et al. | Non-paranoid | 41 | 55 | | 29% | BVAQ | DSM-4 | - | 6 |
| 2021 | Lee et al. | Non-paranoid | 22 | 22 | | 55% | TAS-20 | - | 59.5  (9.90) | 5 |
| 2021 | Luo et al. | Non-paranoid | 135 | 73 | | 39% | TAS-20 | DSM-5 | 50.3  (10.08) | 6 |
| 2021 | Opoka et al. | Non-paranoid | 60 | 40 | | 63% | TAS-20 | M.I.N.I | 51.25  (10.36) | 6 |
| 2021 | Eddy & Hansen | Community | 297 | - | | 85% | TAS-20 | - | 45.84 (11.29) | 0 |
| 2020 | Ma et al. | Community | 2626 | - | | 61% | TAS-20 | - | 54.77  (6.82) | 0 |
| 2020 | Ustundag et al. | Non-paranoid | 208 | - | | 39% | TAS-20 | DSM-4 | - | 4 |
| 2020 | Yang et al. | Community | 552 |  | | 43% | TAS-20 | - | - | 0 |
| 2020 | Vakhrusheva et al. | Non-paranoid | 53 | 19 | | 40% | TAS-20 | DSM-4 | 50.54  (14.90) | 7 |
| 2019 | Caccamo et al. | Non-paranoid | 73 | - | | 48% | TAS-20 | ICD-10 | - | 1 |
| 2019 | Demirkol et al. | Non-paranoid | 113 | - | | 52% | TAS-20 | DSM-5 | - | 3 |
| 2019 | Etchepare et al. | Non-paranoid | 94 | 120 | | 21% | BVAQ | DSM-5 | - | 6 |
| 2019 | Gaweda & Krezolek | Paranoid  100% | 60 | - | | 48% | TAS-20 | DSM-4 | 51.15  (12.11) | 3 |
| 2019 | Ospina et al. | Non-paranoid | 45 | 50 | | 47% | TAS-20 | DSM-4 | 52.12  (12.34) | 6 |
| 2019 | Pozza | Community | 147 | - | | 47% | TAS-20 | - | - | 0 |
| 2019 | Gupta & Gupta | Non-paranoid | 60 | - | | 50% | TAS-20 | ICD-10 | 64  (8.20) | 2 |
| 2018 | Metzner et al. | Paranoid  67% | 21 | - | | 44% | TAS-20 | ICD-10 | 48.76  (12.47) | 2 |
| 2018 | Rehman et al. | Community | 402 | - | | 72% | TAS-20 | - | - | 2 |
| 2018 | Rehman et al. | Community | 401 | - | | 77% | TAS-20 | - | 48.08  (11.55) | 3 |
| 2018 | Kumar et al. | Non-paranoid | 30 | 30 | | - | TAS-20 | ICD-10 | - | 6 |
| 2017 | Lysaker et al. | Non-paranoid | 65 | - | | 4% | TAS-20 | DSM-4 | 58  (11.85) | 5 |
| 2017 | Fung et al. | Community | 212 | - | | 69% | TAS-20 | - | - | 1 |
| 2016 | Kimhy et al. | Non-paranoid | 87 | 50 | | 37% | TAS-20 | SIPS | - | 7 |
| 2016 | Martin et al | Community | 664 | - | | 49% | TAS-20 | - | - | 0 |
| 2016 | Tang et al. | Non-paranoid | 94 | 54 | | 36% | TAS-20 | DSM-4 | 64.54  (11.72) | 7 |
| 2015 | Aaron et al. | Community | 139 | - | | 68% | TAS-20 | - | - | 1 |
| 2015 | van der Velde et al. | Non-paranoid | 38 | 109 | | 24% | BVAQ | DSM-4 | - | 7 |
| 2014 | Fogley et al. | Non-paranoid | 65 | - | | 6% | TAS-20 | DSM-4 | 58.26  (11.43) | 4 |
| 2014 | Koven | Community | 96 | - | | 60% | TAS-20 | - | - | 0 |
| 2013 | De Berardis et al. | Non-paranoid | 30 | - | | 57% | TAS-20 | DSM-4 | 52.25  (11.94) | 7 |
| 2013 | Rus-Calafell et al. | Community | 98 | - | | 67% | TAS-20 | - | - | 0 |
| 2013 | Swart et al. | Non-paranoid | 18 | 18 | | 20% | TAS-20 | DSM-4 | - | 6 |
| 2013 | Lysaker et al. | Non-paranoid | 65 | - | | 5% | TAS-20 | DSM-4 | 57.34  (13.88) | 4 |
| 2013 | Chung et al. | Non-paranoid | 24 | - | | 21% | TAS-20 | DSM-4 | 60.83  (10.15) | 2 |
| 2012 | Dickey et al. | Non-paranoid | - | - | | - | BVAQ | DSM-4 | - | 0 |
| 2012 | Kimhy et al. | Non-paranoid | 44 | 20 | | 36% | TAS-20 | DSM-4 | - | 6 |
| 2012 | Kubota et al. | Non-paranoid | 44 | 44 | | 41% | TAS-20 | DSM-4 | 57.34  (0.08) | 7 |
| 2012 | Picardi et al. | Non-paranoid | 146 | - | | 57% | TAS-20 | DSM-4 | - | 3 |
| 2012 | Son et al. | Non-paranoid | 68 | - | | 44% | TAS-20 | DSM-4 | 55.60  (13.60) | 5 |
| 2011 | Dimaggio et al. | Non-paranoid | 20 | 35 | | 50% | TAS-20 | DSM-5 | - | 4 |
| 2011 | Heshmati et al. | Non-paranoid | 180 | - | | - | TAS-20 | - | - | 2 |
| 2011 | Kubota et al. | Non-paranoid | 21 | 24 | | 33% | TAS-20 | DSM-4 | 58.00  (7.90) | 7 |
| 2011 | Preti et al. | Community | 256 | - | | 54% | TAS-20 | - | - | 2 |
| 2011 | Yu et al. | Paranoia  100% | 60 | 60 | | 50% | TAS-20 | ICD-10 | - | 5 |
| 2011 | Seghers et al. | Community | 72 | - | | 57% | TAS-20 | - | - | 0 |
| 2011 | van Rijn et al. | High risk | 34 | 23 | | 32% | TAS-20 | DSM-4 | - | 5 |
| 2010 | Henry et al. | Non-paranoid | 29 | 30 | | 52% | BVAQ | DSM-4 | - | 6 |
| 2010 | Heshmati et al. | Non-paranoid | 60 | 140 | | - | TAS-20 | - | 60.46  (10.94) | 5 |
| 2010 | Koelkebeck et al. | Non-paranoid | 23 | 23 | | 48% | TAS-20 | DSM-4 | 48.20  (7.30) | 5 |
| 2009 | van der Meer et al. | Non-paranoid | 31 | 44 | | 33% | BVAQ | DSM-4 | - | 6 |
| 2008 | Laroi et al. | Community | 107 | - | | 54% | BVAQ | - | - | 0 |
| 2008 | Serper & Berenbaum | Non-paranoid | 34 | - | | 21% | TAS-20 | DSM-4 | - | 4 |
| 2008 | Valdes et al. | Paranoid  100% | 37 | 37 | | 33% |  | DSM-4 | 39.63  (14.90) | 6 |
| 2007 | van't Wout et al. | Paranoia  67% | 43 | 44 | | 44% | BVAQ | DSM-4 | - | 5 |
| 2005 | Todarello et al. | Non-paranoid | 29 | - | | 24% | TAS-20 | - | 65.8  (9.90) | 3 |
| 2004 | Maggini & Raballo | Paranoid  56% | 128 | - | | 32% | TAS-20 | DSM-4 | 55.28  (17.42) | 1 |
| 2004 | van't Wout et al. | Community | 40 | - | | 44% | BVAQ | - | - | 2 |
| 2003 | Maggini et al. | Paranoid  53% | 76 | - | | 38% | TAS-20 | DSM-4 | - | 3 |
| 2002 | Maggini et al. | Paranoid  81% | 57 | - | | 37% | TAS-20 | DSM-4 | 52.46  (17.38) | 3 |
| 2001 | Cedro et al. | Paranoid  100% | 50 | 50 | | 50% | TAS-20 | DSM-4 | 52.3  (13.5) | 5 |
| 2000 | Solano et al. | Non-paranoid | 20 | - | | 50% | TAS-20 | DSM-4 | 64.20  (9.20) | 3 |
| 1995 | Stanghellini & Ricca | Non-paranoid | 20 | - | | 60% | TAS-20 | DSM-3 | - | 3 |
| 1993 | Prince & Berenbaum | Community | 119 | - | | 45% | TAS-20 | - | - | 2 |
